# Construction and validation of an instrument for event-related sterility of processed healthcare products

**DOI:** 10.1590/0034-7167-2024-0021

**Published:** 2024-09-06

**Authors:** Vanessa Aparecida Vilas-Boas, Louise Assumpção Rondini, Thamiris Cavazzani Vegro Czempik, Ada Helena Melo Lorenzetti, Kazuko Uchikawa Graziano, Ariane Polidoro Dini

**Affiliations:** I Universidade Estadual de Campinas. Campinas, São Paulo, Brazil; II Universidade Estadual de Campinas, Hospital da Mulher Prof. Dr. José Aristodemo Pinotti. Campinas, São Paulo, Brazil; III Universidade de São Paulo. São Paulo, São Paulo, Brazil

**Keywords:** Sterilization, Time Factors, Validation Study, Nursing Assessment, Equipment and Supplies, Esterilización, Fecha de Caducidad de Productos, Estudio de Validación, Evaluación en Enfermería, Almacenamiento de Productos

## Abstract

**Objective:**

To construct and validate an instrument to assess events related to maintaining the sterility of processed healthcare products.

**Methods:**

This methodological study developed the instrument through analysis by a panel of experts, focusing on the integrity of commonly used packaging: spunbond-meltblown-spunbond and medical-grade paper. The instrument was analyzed using the Content Validity Index and Content Validity Ratio (≥ 0.80) and modified Kappa (≥ 0.74). The instrument underwent pre-testing.

**Results:**

Six experienced professionals participated in the expert panel. After two rounds, the final version of the instrument contained five dimensions. In the pre-test, 30 nursing professionals participated, of whom 86.67% considered the instrument good, and 90% found it understandable.

**Conclusion:**

The construction and validation followed literature recommendations. The instrument is available, aiding in the safe use of processed healthcare products.

## RESUMEN

**Objetivo:**

Construir y validar instrumento para evaluar evento relacionado a manutención de la esterilidad de productos para salud procesados.

**Métodos:**

Estudio metodológico para desarrollar instrumento mediante análisis por comité de jueces. Considerado como aspecto rector la integridad de envases más utilizados en la práctica: de no tejido y de papel grado quírurgico. Realizado análisis por Índice de Validez de Contenido y Razón de Validez de Contenido ≥ 0,80 y Kappa modificado ≥ 0,74. Sometido el instrumento a pre-prueba.

**Resultados:**

Participaron del comité de jueces seis profesionales experientes en la temática. Trás dos rondas, fue constituída la versión final del instrumento, conteniendo cinco dimensiones. En la pre-prueba, participaron 30 profesionales de enfermería, en que 86,67% consideraron el instrumento bueno; y 90%, comprensible.

**Conclusion:**

La construcción y validación siguió las recomendaciones de la literatura. El instrumento está disponible para ser utilizado, auxiliando el uso seguro del producto para salud.

## INTRODUCTION

Healthcare-associated infections affect millions of people worldwide, constituting a public health problem. In particular, surgical site infection is common and considered an avoidable adverse event. Recently, the World Health Organization proposed seven strategic objectives to achieve by 2030. Among them, Objective 3 advocates for ensuring the safety of all clinical processes, which involves implementing rigorous measures for infection prevention and control and the safe use of medical devices^(1)^.

Events related to maintaining the sterility of healthcare products are those that can damage the integrity of the packaging or sealing of the product due to adverse environmental or behavioral storage conditions^(2)^. Such damage can be harmful as it predisposes the healthcare products to contamination. Thus, sterility control should be enhanced by analyzing the events that occurred with the healthcare products, as contamination can occur at any time, including immediately after processing^(2, 3, 4)^. However, many institutions still opt for an arbitrary sterilization expiration date without rigorous and systematic evaluation of these events.

The expiration date of healthcare products processed by the Central Sterile Services Department (CSSD) was historically based on studies from the 1970s using microbiological tests on sterilized products under adverse storage conditions. The authors considered contamination from the third day after sterilization for products packaged in single-wrap muslin and 28 days in double-wrap muslin on open shelves, maintaining sterility for 63 days or more when using a cover bag^(5)^. Only from 1984 was it considered that the expiration date in previous studies was based on time and not on other variables such as temperature, local humidity, transportation, air movement, and packaging, which are properties capable of contaminating a material^(6)^. Thus, a concept of event-related sterility emerged, opposing the time-related sterility paradigm.

Over the years, it has become evident that the storage time of healthcare products, as long as it is within recommendations, does not affect the material’s susceptibility to contamination even when deliberately exposed to microorganisms^(3, 7)^. In the 19th century, Louis Pasteur proved that sterile samples only proliferate microorganisms through contact with already contaminated locations^(8)^.

Following this logic, sterilized products maintained with intact packaging and sealing remain sterile until an event or damage to the package integrity occurs. This is because sterilization packaging systems should ensure safety through the following characteristics: barrier to microorganism entry, hermetic sealing, protection of the package content against physical damage, and resistance to punctures and tears^(7)^. For example, medical-grade paper and non-woven spunbond-meltblown-spunbond (SMS) have been widely used for healthcare products sterilization because of their low cost, excellent antimicrobial barrier, high permeability to sterilizing agents, and good mechanical resistance^(7)^.

The CSSD is responsible for processing healthcare products, thus performing the following functions: cleaning, inspection, packaging, sterilization, and distribution to care units^(9)^. However, even if all steps conducted within the CSSD meet quality and safety standards, the sterility of the processing can be compromised if the care units do not handle, transport, and store healthcare products carefully and neglect the quality of the sealing and packaging of processed healthcare products^(2, 10)^.

A Brazilian study conducted with 11 large hospitals found a lack of knowledge among nursing professionals about the healthcare products processing stages, types of packaging used, storage care, and technical regulations^(10)^. Important aspects evaluated by this and other studies reinforce the inadequate storage and transportation of sterilized healthcare products, lack of packaging inspection before the sterilization process and before use, and absence of checking the color change of the exposure chemical indicator, among others^(2, 7, 10)^.

Therefore, more recent studies relate the loss of healthcare products sterility to events rather than a specific date^(2, 3, 7)^. The Collegiate Board Resolution (RDC) No. 15/2012, Article 4, Item VII adheres to the principles of event-related sterility^(9)^. However, while describing the need for a packaging integrity assessment plan, it does not clarify how to develop or implement this plan, leading to a lack of uniformity in practices across different institutions in the country and among regulatory bodies in each region.

After an in-depth literature review on the subject, it was found no valid instrument to assess events at the packaging of sterilized healthcare products. Given maintaining sterility depends on various factors and methods to implement this safety principle are still scarce, this study constructed and validated the instrument “Assessment of Event Related to Maintaining the Sterility of Healthcare Products” (AERMS). This instrument can integrate the packaging integrity assessment plan and stop using healthcare products in risky situations, especially those critical for invasive procedures.

## OBJECTIVE

To construct and validate an instrument to evaluate events related to maintaining the sterility of healthcare products processed by the CSSD, focusing on the integrity of SMS and medical-grade paper packaging.

## METHODS

### Ethical aspects

The Research Ethics Committee approved this study, which followed the recommendations of Resolution No. 466/2012 of the National Health Council. Participants signed the Informed Consent Form in two copies.

### Design, period, and study location

This methodological study involves developing instruments. It investigates methods for obtaining, organizing, and analyzing data based on the development, validation, and evaluation of research instruments and techniques. The report was based on the COnsensus-based Standards for the selection of health Measurement INstruments (COSMIN)^(11)^.

The study was conducted in two stages between October 2021 and December 2022. The first stage involved constructing the instrument, and the second involved its evaluation by a panel of experts for content validity^(11, 12)^. The pre-test was conducted in a medium-sized tertiary/quaternary public teaching hospital in the interior of São Paulo state.

### Population and inclusion and exclusion criteria

The target population comprised nursing professionals working in the CSSD and user units with non-probabilistic convenience sampling. The criteria are presented below (Chart 1).

**Chart 1:** Selection criteria for participants, Brazil, 2021

CRITERION 1	- Must have a doctorate, master’s, and/or specialization in the specific area. - Must have practical and/or teaching and/or research experience of at least three years in the specific area, either nationally covering different regions of the country or internationally (requires proficiency in Portuguese). Additionally, they must meet at least one of the following: - Participate or have participated for at least two years in a research group in the area. - Participate in scientific events related to the theme. - Have published articles in reference journals.
CRITERION 2	- Must be a nurse and/or nursing technician at the study institution. - Must have at least three years of practical experience in the nursing field.

For content validation, the constructed instrument was submitted to a panel of experts in the area for the removal, modification, or addition of items^(11)^. The research team created a list of professionals with expertise in the topic, and the selection was based on an analysis of their Lattes Curriculum. The panel was expected to consist of 5 to 20 experts, and the pre-test was applied to a sample of 30 to 40 individuals from the target population^(12)^.

### Study protocol

#### Stage 1: Instrument construction

The instrument construction was based on literature, national guidelines^(13, 14)^, international guidelines^(7, 15, 16, 17)^, Collegiate Board Resolution (RDC) No. 15 of March 15, 2012, which addresses good practices for processing healthcare products in Brazil^(9)^, research team formation, institutional protocols, in loco observation of work processes, and nursing professionals’ experience.

As an initial step, a search was conducted in the Bireme, PubMed, Embase, Cochrane, CINAHL, and SciELO databases to: a) establish the conceptual structure, b) verify existing instruments, c) define the instrument’s objectives, and d) define the population to be involved, determining the construct of interest and its dimensions. Key events for the instrument’s composition were identified, focusing on healthcare products packaged in SMS or medical-grade paper. Subsequently, items and dimensions were constructed, response scales selected, and items organized, structuring the instrument^(12)^.

A preliminary version of the instrument was created with the assistance of CSSD nursing staff at the study institution using the brainstorming technique, to stimulate creativity by generating numerous ideas on a subject^(18, 19)^. Three brainstorming sessions were held between March and April 2022.

Next, to verify if the items in each domain were appropriate and if the instrument could be applied in different realities of public and private hospitals with low and high demands, face validation was conducted with three other health institutions using the benchmarking strategy in a focus group^(20, 21, 22)^. The focus group was conducted online in a single 60-minute session. Researchers presented the instrument to the participants, who were then allowed to express their opinions on its usefulness in their hospital context, clarify doubts, or suggest modifications. Based on the suggestions, the experts received the first version of the instrument. An instruction guide was also developed to facilitate instrument completion, containing definitions of each addressed item.

#### Stage 2: Content validation

Data for the second stage were collected between June and December 2022. The experts’ evaluation was sequential, individual, and anonymous, with commented feedback, repeated until a consensus was reached. The invitation letter was sent via email, considering a single attempt for a response. The clarity and relevance of the items, dimension comprehensiveness, and overall appearance of the instrument were evaluated^(12)^. Experts used a four-point Likert scale, where “1” meant “item not necessary or relevant/unclear/not comprehensive,” and “4” meant “item definitely essential, highly relevant/clear/comprehensive”^(20)^.

After this phase, a pre-test was conducted for semantic and practical evaluation. Participants received the instrument, a completion guide, and a form containing: a) personal data for sample characterization, b) overall instrument evaluation (good, fair, or poor) and whether the questions align with the institution’s values and professional practices, c) specific evaluation of the instrument’s instructions, items, and responses comprehension, besides assessing whether each item was considered important, and d) completion time (start/end).

### Results analysis and statistics

Data were entered into a Google Drive spreadsheet, and results were analyzed by the researchers between each round. To measure agreement among experts, the Content Validity Index (CVI), Content Validity Ratio (CVR), and modified Kappa (k) coefficient were used, developed for content validity studies^(21)^. The CVI/CVR was calculated by the number of “3” and “4” responses divided by the total number of responses, indicating the proportion of experts agreeing on specific aspects of the instrument and its items, with an index ≥ 0.80 considered for this study^(12)^. For the modified Kappa, values from 0.40 to 0.59 were considered reasonable; 0.60 to 0.74 good; and above 0.74 excellent^(23)^.

Items that did not reach the required score in both tests were reformulated or excluded, and the experts re-evaluated the instrument, resulting in the final instrument after the second round. For the pre-test, descriptive statistics were used, including percentage, mean, median, standard deviation (SD), and percentiles 25 and 75 (Q1 and Q3).

## RESULTS

The preliminary version of the instrument was constructed with 51 items and five dimensions: 1) Product presentation, 2) Event related, 3) Packaging sealing, 4) Chemical indicator, and 5) Occurrences.

For face validation, four nurses from public and private institutions in São Paulo state and one nursing technician from the study institution were invited. Three nurses participated in the focus group: one from a private hospital and one from a public hospital in two municipalities in the interior of São Paulo state; and a nurse working in two hospitals, one public and one private, in a municipality in Rio de Janeiro state. Two nurses had specialization in CSSD management, and one had over three years of practical experience in CSSD, meeting the inclusion criteria.

After presenting the instrument, participants judged the items as appropriate for evaluating event-related sterility of processed healthcare products. For practical application according to each institution’s reality, it was suggested to divide the product evaluation into three sessions: “preparation,” “storage and distribution,” and “anytime.” The traffic light model with green, yellow, and red colors was used respectively for each session, leading to the first version of the instrument (supplementary material).

Content validation occurred over three months, between the initial invitation and the experts reaching a consensus on all evaluated items. To form the expert panel, 25 eligible professionals were invited based on established criteria. Ten responded, with one declining and nine agreeing to participate. Of those, five completed the instrument evaluation in the first round, with a 55.56% response rate (5/9). In the second round, these five experts received back the reformulated instrument, with four responding. Four additional professionals were invited to join the panel, with one accepting. Thus, the second-round response rate was 83.33% (5/6).

Five Brazilian experts and one foreign expert fluent in Portuguese constituted the panel, including three from São Paulo state, one from the Federal District, and two residing abroad. All had postgraduate degrees: one with a master’s degree, two with doctorates, and three with post-doctorates. Their areas of expertise included regulatory bodies, teaching and research, and hospital assistance, with 3 to 35 years of experience (Mean = 17.33; SD = 11.33).

After the first round, the items “Packaging: single or double,” “Preparation identification label: yes or no,” “Package to be distributed to units presents,” and “Twist” were excluded. The item “Initials/Stamp” was reformulated to “Professional responsible for verification.” The item “Pen markings directly on the packaging” met the expected value; however, based on expert suggestions, it was reclassified as an event in Dimension 2. All instrument dimensions were considered comprehensive (CVI/CVR = 1.00; modified Kappa = 1.00).

In evaluating the completion guide, the dimensions were analyzed for relevance, clarity, and comprehensiveness (Table 2). The overall appearance evaluation of the completion guide obtained a CVI = 0.80 and modified Kappa = 0.76, considered excellent.

**Table 2:** Analysis of the first round of the instrument completion guide Assessment of Event Related to Maintaining the Sterility of Healthcare Products, Brazil, 2022

Dimensions	Pertinence		Clarity		Comprehensiveness	
	CVI*/CVR^†^	k^‡^	CVI*	*k* ^‡^	CVI^*^	*k* ^‡^
Presentation of the product	1.00	1.00	0.80	0.76	0.80	0.76
Event related	1.00	1.00	1.00	1.00	1.00	1.00
Packaging sealing	1.00	1.00	1.00	1.00	0.80	0.76
Chemical indicator	1.00	1.00	1.00	1.00	1.00	1.00
Occurrences	1.00	1.00	1.00	1.00	1.00	1.00

*CVI – Content Validity Index; † CVR – Content Validity Ratio; ‡ k – Modified Kappa Coefficient.

With the adjustment of the items, the instrument was no longer subdivided into sections and was presented in a single column in the second version. The item “Product Identification” was added, referring to the product name or any identification contained on the label, different from the “Order No.,” which refers to the number of evaluations performed. The instruction “Evaluate items 2 to 5. If any nonconformity is identified, DO NOT use the package. Return it to the CSSD for evaluation” was also added to guide the evaluator on how to proceed.

After formatting the instrument with the alteration, exclusion, and addition of items, it was submitted to a second evaluation by the experts, where all items and the overall appearance of the instrument achieved CVI/CVR ≥ 0.80 and modified Kappa ≥ 0.76, with no alterations to its content and structure (supplementary material).

The pre-test was conducted from October to December 2022. Thirty nursing professionals participated, with 33.33% reporting completed high school education, 16.67% completed higher education, and 50% postgraduate education. Regarding their role in the institution, 56.67% were nursing technicians, and 43.33% were nurses, of whom 20% held management positions. The average age was 42.97 years (SD = 7.34) and the average professional experience was 16.03 years (SD = 6.77). The instrument was tested throughout the hospital, with 33.33% in the CSSD and 66.67% in the user units (operating block, intensive care units, inpatient unit, outpatient clinic, and emergency department) before using the healthcare product or in internal audit processes.

Completion time varied from 1 to 13 minutes (Mean = 4.33; SD = 2.95; Median = 4.00; Q1 = 2.00; Q3 = 5.00). In the overall evaluation of the instrument, 86.67% of participants considered it good, and 90.00% stated that it aligns with the institution’s values and professional practices. In the specific evaluation of the items, 86.67% partially (20.00%) or totally (66.67%) agreed that it was easy to understand the instrument’s instructions and items; 90.00% partially (26.67%) or totally (63.33%) agreed that it was easy to understand and mark the instrument’s responses.

The items participants reported difficulty understanding included: Order No., Delamination, Burn, Crease (tunnel), and Re-evaluation after a fall. Following the suggestions, “Order No.” was replaced with “Number of product evaluations”; in “Re-evaluation after a fall,” “If yes, re-evaluate after fall” was added; and in this topic, “Not applicable” was replaced with “No damage to package integrity.” For the remaining items pointed out, it was found that participants did not consult the guide during completion.

After the pre-test, since there were no content alterations, the instrument did not need to return to expert evaluation, and the content was considered valid for evaluating event-related sterility of healthcare products (supplementary material).

**Table 1:** Analysis of the first round of the instrument Assessment of Event Related to Maintaining the Sterility of Healthcare Products, Brazil, 2022

Sessions/Dimensions/Items	Pertinence		Clarity	
	CVI*/CVR^†^	*k* ^‡^	CVI*	*k* ^†^
Order No.	0.80	0.76	0.80	0.76
PREPARATION	1.00	1.00	1.00	1.00
1. Presentation of the product	1.00	1.00	0.80	0.76
Packaging (single/double)	0.60	0.42	1.00	1.00
Protection in case of healthcare products puncture and cuts	1.00	1.00	0.80	0.76
Preparation identification label containing: product name, number of pieces, preparation date, presence of indicator, and preparer’s name	0.60	0.42	0.80	0.76
Sterilization identification label containing: product name, number of pieces, batch or load number, sterilization date, expiration date, sterilization method, and responsible person’s name	0.80	0.76	0.80	0.76
Pen markings directly on the packaging	0.80	0.76	1.00	1.00
Initials/Stamp	1.00	1.00	0.80	0.76
Date	1.00	1.00	0.80	0.76
STORAGE AND DISTRIBUTION	0.80	0.76	1.00	1.00
2. Event related	0.80	0.76	1.00	1.00
Package to be distributed to units presents:	0.60	0.42	1.00	1.00
Tear	0.80	0.76	1.00	1.00
Cut	0.80	0.76	1.00	1.00
Twist	0.60	0.42	0.80	0.76
Punctures/Micropunctures (look against the light for medical-grade paper)	1.00	1.00	1.00	1.00
Stains on the packaging or healthcare products^±^	1.00	1.00	0.80	0.76
Moisture on the packaging or healthcare products^±^	1.00	1.00	0.80	0.76
Dirt on the packaging or healthcare products^±^	1.00	1.00	0.80	0.76
3. Packaging sealing	1.00	1.00	1.00	1.00
Sealing presents:	0.80	0.76	1.00	1.00
Adhesion failure	0.80	0.76	0.80	0.76
3.1. For medical-grade paper	1.00	1.00	1.00	1.00
Sealing presents:	1.00	1.00	1.00	1.00
Bubble	1.00	1.00	1.00	1.00
Delamination	1.00	1.00	1.00	1.00
Burn	0.80	0.76	1.00	1.00
Fold or crease	1.00	1.00	1.00	1.00
4. Chemical indicator	1.00	1.00	0.80	0.76
Colored	1.00	1.00	0.80	0.76
Absent	1.00	1.00	0.80	0.76
Color change failure	1.00	1.00	0.80	0.76
Initials/Stamp	0.80	0.76	0.80	0.76
Date	1.00	1.00	1.00	1.00
5. ANYTIME	1.00	1.00	0.80	0.76
Occurrences	1.00	1.00	1.00	1.00
Suspected that the package has been opened	1.00	1.00	1.00	1.00
Expired expiration date	1.00	1.00	1.00	1.00
Package fell to the ground	1.00	1.00	0.80	0.76
Re-evaluation after fall	1.00	1.00	1.00	1.00
Tear	1.00	1.00	0.80	0.76
Cut	1.00	1.00	0.80	0.76
Twist	1.00	1.00	0.80	0.76
Punctures/Micropunctures	1.00	1.00	1.00	1.00
Stains	1.00	1.00	1.00	1.00
Moisture	1.00	1.00	0.80	0.76
Dirt	1.00	1.00	1.00	1.00
Not applicable	1.00	1.00	0.80	0.76
Initials/Stamp	0.80	0.76	0.60	0.42
Date	1.00	1.00	0.80	0.76

*CVI – Content Validity Index; † CVR – Content Validity Ratio; ‡ k – Modified Kappa Coefficient.

## DISCUSSION

Scientific evidence indicates sterility is compromised by events that damage packaging and sealing integrity^(17, 13, 14, 15, 16, 17)^. As a checklist, the AERMS evaluates event-related occurrences in processed healthcare products, interrupting their use in risky situations, especially critical healthcare products for invasive procedures.

During instrument construction, the active contribution of the nursing team aimed to improve the acceptability, relevance, and quality of the evaluation, as researchers’ perspectives may not align with service users’ priorities^(24)^. Benchmarking in healthcare measures and compares results from other institutions to implement the best practices^(22)^. Initially, the AERMS was designed to be used at different processing stages, from product preparation to patient use, considering checks performed by different people at different times within the same form. Through content validation, it was realized this structure would hinder implementation, and because of the exclusion of some items, there would be no justification to maintain fragmented inspection moments. Therefore, the instrument can adapt to service realities, whether for inspecting healthcare products stored in the CSSD or user units, for audit purposes, or in occurrences with sterilized healthcare products, but it should primarily be incorporated as a routine and automatic practice.

Content validity is defined as the degree of relevance and representativeness of the addressed items about the instrument’s specific purpose^(21)^. For this type of validation, there is still no consensus in the literature on the number of experts to be involved^(1, 25)^. However, it is recommended to have at least five people for statistical data analysis procedures, considering the instrument’s characteristics and the number of items, as well as the formation, experience, and availability of the experts on the study subject^(11, 12)^. Although nine eligible individuals agreed to participate by signing the Informed Consent Form, only five returned with the instrument evaluation, maintaining the minimum established limit in both rounds. It is noted that healthcare product processing is still a field with few specialists, justifying the response rate obtained in the AERMS content validation.

Regarding the excluded items for “Packaging: single or double,” it is understood that, depending on the packaging type, the evaluator may not distinguish between primary and secondary packaging. Concerning the “Preparation identification label,” not all services adopt this practice; however, RDC No. 15/2012, Article 85, Item VI mandates the preparer’s name^(9)^. The item “Package to be distributed to units presents” was excluded, considering the instrument’s applicability at any time, not just during CSSD product distribution. The term “Twist” was excluded because of its ambiguity with the concept of fold. A study in France indicates contamination risk resulting from the folding of medical-grade paper in primary packaging of healthcare products stored for at least six months^(4)^.

For the remaining items, there was a consensus on their relevance and clarity, especially regarding packaging integrity and maintaining sterility. Although the paradigm shift from time-related to event-related sterility is advocated, the item “Expired expiration date” was maintained, considering Brazil is in a transition process, and many institutions still use the sterilization expiration date recommended by RDC No. 15/2012^(9)^.

This expiration date can aid in managing the stock rather than controlling the healthcare product sterility loss. An inactive product in stock represents a financial resource waste that could be invested in necessary supplies^(26)^. Unnecessary healthcare products processing due to expired expiration dates incurs costs to the institution, staff wear, premature material wear, increased packaging waste, and waste of natural resources such as water and electricity. A study conducted in a tertiary hospital in Goa, India, calculated that the monthly cost of sterilizing expired healthcare products was 8,772 Indian rupees, equivalent to about 105 dollars today^(26)^. Material and human resource management in the CSSD is critical to the health system, as operational excellence directly influences user and patient care. It aligns with sustainable development goals (SDGs), particularly SDG-3 Health and Well-being; SDG-9 Industry, Innovation, and Infrastructure; and SDG-12 Responsible Consumption and Production. Therefore, the “Expired expiration date” item in the AERMS can be used as an indicator of healthcare product turnover based on an inspection protocol implementation.

In the present study, once the instrument was evaluated and consensus reached among experts, it was subjected to a pre-test with a small sample to verify if all items were comprehensible to the target population^(12)^. This is an important research stage applied in recent content validation studies^(1, 27)^ to help researchers identify potential problems or biases during data collection and verify the instrument’s applicability in real life. The pre-test allowed quick AERMS completion with comprehension percentages equal to or higher than 86% for the items, aligning the instrument with the institution’s values and practices. However, participants were found to consult the completion guide less, a separate document, with one copy per sector for consultation. After the pre-test, the authors chose to keep the instructions on the back of the instrument for quick reference as needed.

Therefore, when using the event-related sterility concept, healthcare institutions must establish good practices to ensure the safe handling, transportation, and storage of sterilized healthcare products, ensuring packaging integrity and reducing waste, workload, and costs^(7, 26, 28)^.

### Study limitations

A limitation was the authors’ decision not to include cotton fabric due to difficulty controlling its quality specifics, such as mending, number of washes, or others, especially when evaluated by users. Other types of packaging like crepe paper and Tyvek® were not included as they are not used in the institution where the study was developed, although the authors believe the available items in the instrument could also be useful for evaluating these packages.

### Contributions to the field of nursing

Considering that maintaining the sterility of healthcare products depends on various factors and that methods to implement this safety principle are still scarce, this study’s contribution was the construction and validation of an instrument to assess events that may compromise packaging integrity. The AERMS can be useful in integrating the packaging integrity evaluation plan and shifting the paradigm from time-related to event-related sterility in clinical practice. This will help managers evaluate their work processes in the CSSD and user units and support healthcare professionals involved in direct care in making decisions about the safe use of healthcare product, contributing to patient safety.

## CONCLUSION

The construction and content validation of the “Assessment of Event Related to Maintaining the Sterility of Healthcare Products” (AERMS) instrument for SMS and medical-grade paper packaging followed literature recommendations and is available for use. This instrument aids in managing work processes and decision-making regarding the safe use of healthcare products.

## Supplementary Material

0034-7167-reben-77-04-e20240021-suppl01

0034-7167-reben-77-04-e20240021-suppl02

0034-7167-reben-77-04-e20240021-suppl03

0034-7167-reben-77-04-e20240021-suppl04

0034-7167-reben-77-04-e20240021-suppl05

## Data Availability

https://doi.org/10.25824/redu/KAXNUY
