# Supplementary material for: Construction and validation of an instrument for event-related sterility of processed healthcare products
Source: Rev Bras Enferm. 2024 Sep 6;77(4):e20240021. doi: 10.1590/0034-7167-2024-0021 (PMC11382677; doi:10.1590/0034-7167-2024-0021)
Supplement: 0034-7167-reben-77-04-e20240021-suppl01 [file 0034-7167-reben-77-04-e20240021-suppl01.pdf]

FORMULÁRIO PARA AVALIAÇÃO DOS JUÍZES REFERENTE AO:

A) Instrumento “Avaliação de produtos para saúde (PPS) esterilizados”

B) Guia de preenchimento

A) Instrumento “Avaliação de produtos para saúde (PPS) esterilizados”

Tem como finalidade obter informações que permitam avaliar a integridade da embalagem dos produtos para saúde (PPS) e/ou pacotes, que foram esterilizados e que estão disponíveis para uso no paciente.

Por favor, avalie os itens e subitens com relação à **pertinência** e à **clareza**.

**Item**	**Pertinência**				**Clareza**			
Nº ordem: __________	1	2	3	4	1	2	3	4
**PREPARO**	1	2	3	4	1	2	3	4
**1. APRESENTAÇÃO DO PRODUTO**	1	2	3	4	1	2	3	4
Embalagem:	1	2	3	4	1	2	3	4
simples	1	2	3	4	1	2	3	4
dupla	1	2	3	4	1	2	3	4
Proteção em caso de PPS perfurocortantes	1	2	3	4	1	2	3	4
sim	1	2	3	4	1	2	3	4
não	1	2	3	4	1	2	3	4
Etiqueta de identificação de **preparo** contendo: nome do produto, número de peças, data do preparo, presença de indicador, nome do preparador	1	2	3	4	1	2	3	4
sim	1	2	3	4	1	2	3	4
não	1	2	3	4	1	2	3	4
Etiqueta de identificação de **esterilização** contendo: nome do produto, número de peças, número do lote ou carga, data da esterilização, data limite de uso, método de esterilização, nome do responsável pela esterilização.	1	2	3	4	1	2	3	4
sim	1	2	3	4	1	2	3	4
não	1	2	3	4	1	2	3	4
Marcações à caneta diretamente na embalagem	1	2	3	4	1	2	3	4
sim	1	2	3	4	1	2	3	4
não	1	2	3	4	1	2	3	4
Rúbrica/Carimbo:	1	2	3	4	1	2	3	4
Data	1	2	3	4	1	2	3	4
**GUARDA E DISTRIBUIÇÃO**	1	2	3	4	1	2	3	4
**2. EVENTO RELACIONADO**	1	2	3	4	1	2	3	4
O pacote a ser distribuído para as unidades apresenta:	1	2	3	4	1	2	3	4
Rasgo	1	2	3	4	1	2	3	4
sim	1	2	3	4	1	2	3	4

não	1	2	3	4	1	2	3	4
Corte	1	2	3	4	1	2	3	4
sim	1	2	3	4	1	2	3	4
não	1	2	3	4	1	2	3	4
Torção	1	2	3	4	1	2	3	4
sim	1	2	3	4	1	2	3	4
não	1	2	3	4	1	2	3	4
Furos/microfuros (olhar contra à luz em caso de papel grau cirúrgico)	1	2	3	4	1	2	3	4
sim	1	2	3	4	1	2	3	4
não	1	2	3	4	1	2	3	4
Manchas na embalagem ou PPS	1	2	3	4	1	2	3	4
sim	1	2	3	4	1	2	3	4
não	1	2	3	4	1	2	3	4
Umidade na embalagem ou PPS	1	2	3	4	1	2	3	4
sim	1	2	3	4	1	2	3	4
não	1	2	3	4	1	2	3	4
Sujidade na embalagem ou PPS	1	2	3	4	1	2	3	4
sim	1	2	3	4	1	2	3	4
não	1	2	3	4	1	2	3	4
**3. SELAGEM DA EMBALAGEM**	1	2	3	4	1	2	3	4
A selagem apresenta:	1	2	3	4	1	2	3	4
Falha na aderência	1	2	3	4	1	2	3	4
sim	1	2	3	4	1	2	3	4
não	1	2	3	4	1	2	3	4
**3.1 Para PAPEL GRAU CIRÚRGICO**	1	2	3	4	1	2	3	4
A selagem apresenta:	1	2	3	4	1	2	3	4
Bolha	1	2	3	4	1	2	3	4
sim	1	2	3	4	1	2	3	4
não	1	2	3	4	1	2	3	4
Delaminação	1	2	3	4	1	2	3	4
sim	1	2	3	4	1	2	3	4
não	1	2	3	4	1	2	3	4
Queimadura	1	2	3	4	1	2	3	4
sim	1	2	3	4	1	2	3	4
não	1	2	3	4	1	2	3	4
Dobra ou Vinco	1	2	3	4	1	2	3	4
sim	1	2	3	4	1	2	3	4
não	1	2	3	4	1	2	3	4
**4. INDICADOR QUÍMICO**	1	2	3	4	1	2	3	4
Corado	1	2	3	4	1	2	3	4
Ausente	1	2	3	4	1	2	3	4
Falha na coloração	1	2	3	4	1	2	3	4
Rúbrica/Carimbo:	1	2	3	4	1	2	3	4
Data	1	2	3	4	1	2	3	4
**A QUALQUER MOMENTO**	1	2	3	4	1	2	3	4
**5. INTERCORRÊNCIAS**	1	2	3	4	1	2	3	4
Suspeita de que o pacote tenha sido aberto	1	2	3	4	1	2	3	4
sim	1	2	3	4	1	2	3	4
não	1	2	3	4	1	2	3	4

Data limite de uso expirada	1	2	3	4	1	2	3	4
sim	1	2	3	4	1	2	3	4
não	1	2	3	4	1	2	3	4
Pacote caiu no chão	1	2	3	4	1	2	3	4
sim*	1	2	3	4	1	2	3	4
não	1	2	3	4	1	2	3	4
REAVALIAÇÃO APÓS QUEDA	1	2	3	4	1	2	3	4
Rasgo	1	2	3	4	1	2	3	4
Corte	1	2	3	4	1	2	3	4
Torção	1	2	3	4	1	2	3	4
Furos/microfuros	1	2	3	4	1	2	3	4
Manchas	1	2	3	4	1	2	3	4
Umidade	1	2	3	4	1	2	3	4
Sujidade	1	2	3	4	1	2	3	4
Não se aplica								
Rúbrica/Carimbo	1	2	3	4	1	2	3	4
Data	1	2	3	4	1	2	3	4
**Comentários do juiz:** se pontuação “1“ou “2” Por favor, faça sugestões.								

Com relação à **abrangência** dos domínios relacionados à **Avaliação de produtos para saúde (PPS) esterilizados**, eles podem ser avaliados como:

Não abrangente	Pouco abrangente	Moderadamente abrangente	Muito abrangente
1	2	3	4

Item	**Comentários do juiz:** se pontuação “1“ou “2” Por favor, faça sugestões.

Guia de Preenchimento do Instrumento “Avaliação de produtos para saúde (PPS) esterilizados”

Tem como finalidade facilitar o preenchimento do instrumento e padronizar os itens a serem monitorados pelos profissionais de saúde, tanto da CME quanto das unidades usuárias. O guia também irá contribuir para o treinamento das equipes nas instituições de saúde.

Por favor, avalie as orientações para o preenchimento e/ou significados/definições dos itens e subitens com relação à **pertinência, clareza e abrangência**.

**Item**	**Pertinência**				**Clareza**				**Abrangência**			
1. APRESENTAÇÃO DO PRODUTO	1	2	3	4	1	2	3	4	1	2	3	4
2. EVENTO RELACIONADO	1	2	3	4	1	2	3	4	1	2	3	4
3. SELAGEM DA EMBALAGEM	1	2	3	4	1	2	3	4	1	2	3	4
4. INDICADOR QUÍMICO	1	2	3	4	1	2	3	4	1	2	3	4
5. INTERCORRÊNCIAS	1	2	3	4	1	2	3	4	1	2	3	4
*Reavaliação após queda	1	2	3	4	1	2	3	4	1	2	3	4
Rúbrica/Carimbo	1	2	3	4	1	2	3	4	1	2	3	4
Data	1	2	3	4	1	2	3	4	1	2	3	4
**Comentários sobre o Guia de Preenchimento do Instrumento:** se pontuação “1“ou “2” por favor, faça sugestões.												

Com relação à **aparência geral do <u>instrumento</u>**, ela pode ser avaliada como:

1	2	3	4

Com relação à **aparência geral do <u>guia de preenchimento</u>**, ela pode ser avaliada como:

1	2	3	4
