# Supplementary material for: Construction and validation of an instrument for event-related sterility of processed healthcare products
Source: Rev Bras Enferm. 2024 Sep 6;77(4):e20240021. doi: 10.1590/0034-7167-2024-0021 (PMC11382677; doi:10.1590/0034-7167-2024-0021)
Supplement: 0034-7167-reben-77-04-e20240021-suppl02 [file 0034-7167-reben-77-04-e20240021-suppl02.pdf]

GUIA DE INSTRUÇÕES PARA PREENCHIMENTO DO INSTRUMENTO

Este guia foi desenvolvido com o objetivo de facilitar o preenchimento do instrumento “Avaliação de evento relacionado à manutenção da esterilidade de produtos para saúde processados (AERME - PPS)”. Isto propiciará uma avaliação padronizada dos Produtos Para Saúde (PPS), durante a guarda e distribuição, e também em seu ponto de uso. Deverá ser utilizado como subsídio para prevenção de infecção na assistência ao paciente e no treinamento para sua utilização.

AVALIAÇÃO DE EVENTO RELACIONADO À MANUTENÇÃO DA ESTERILIDADE DE PRODUTOS PARA SAÚDE PROCESSADOS (AERMS)

Quantidade de avaliações do produto ________: número fornecido ao impresso no momento da avaliação da embalagem, para quantificar o número de avaliações realizadas.

Identificação do produto __________________: nome do produto e/ou qualquer outra identificação presente na etiqueta de identificação da esterilização que permita rastrear ou comparar o impresso e o PPS.

1. **APRESENTAÇÃO DO PRODUTO:** itens que espera-se que o PPS tenha.
   - **Proteção em caso de PPS perfurocortantes:** os PPS perfurocortantes (como tesouras, por exemplo), acondicionados em papel grau cirúrgico, deverão ter um dispositivo de proteção como o silicone por exemplo, ou um pequeno invólucro confeccionado com o próprio papel grau cirúrgico colocado na extremidade do item, com o objetivo de proteger a embalagem principal de perfurações e rasgos. Marcar se há ou não proteção na extremidade perfurocortante.
   - **Etiqueta de identificação da <u>esterilização</u> contendo: nome do produto, número de peças, número do lote ou carga, data da esterilização, data limite de uso, método de esterilização, nome do responsável pela esterilização:** etiqueta autocolante fixada na parte externa da embalagem contendo essas informações. Esta identificação é necessária para o rastreamento da esterilização no caso de alguma intercorrência ou falha de processo. Segue as recomendações da RDC nº 15/2012. Marcar se a etiqueta está presente (sim) ou ausente (não). Se o PPS possuir uma etiqueta, porém sem os dados acima, marcar a opção “não”.

2. **EVENTO RELACIONADO:** falhas de processo ou intercorrências que podem provocar o rompimento da integridade do sistema de embalagem e, consequente, a perda da

esterilidade do PPS. Marcar as opções sim ou não para indicar a ocorrência de cada um dos eventos abaixo descritos.

- **Rasgo/corte:** abertura numa superfície que se rompeu ou dilacerou; separação ou divisão por meio de instrumento cortante.
- **Dobra:** Dobrar, inclinar, vergar, encurvar. Nesse caso, é importante que o profissional saiba avaliar se houve ruptura, fissura e/ou separação da superfície da embalagem que prejudique a esterilidade do produto. Considerar que o papel grau cirúrgico amassa devido a sua manipulação, e também no processo de esterilização.
- **Furos/microfuros:** perfurações. Abertura produzida por lesão externa. Orifício, buraco, abertura. Para embalagens do tipo papel grau cirúrgico, torna-se válida a visualização contra a luz para identificação destes sinais ou outras imperfeições.
- **Marcações à caneta diretamente na embalagem:** todas as informações sobre o PPS e seu processamento devem estar contidas nas etiquetas de preparo e esterilização. A marcação à caneta ou outro tipo de tinta diretamente na embalagem do PPS proporciona risco de dano à embalagem e transferência de tinta para o PPS. Marcar sim ou não para indicar a presença ou ausência de marcações à caneta diretamente na embalagem, respectivamente.
- **Umidade na embalagem ou PPS:** característica ou estado do que está úmido – que está envolto pelo vapor de água ou sutilmente molhado.
- **Sujidade/mancha na embalagem ou PPS:** Marca de cor diferente presente na superfície da embalagem ou do PPS. Sujidade refere-se à qualidade do que é sujo, imundície, sujeira. As manchas são marcas deixadas por sujidade ou nódoa.

3. **SELAGEM DA EMBALAGEM:** processo pelo qual é feito o fechamento das embalagens para esterilização, de forma hermética, garantindo a penetração do agente esterilizante e impedindo a entrada de microrganismos, sujidade e outros agentes contaminantes. No caso do *Spunbond Meltblown Spunbond* (SMS), a selagem é feita com fita adesiva, sendo importante estar bem aderida ao tecido, evitando a exposição do PPS esterilizado ao ambiente e sua consequente contaminação. Para papel grau cirúrgico, a selagem é feita com termoseladora, conforme Art.80 da RDC nº 15 de 15 de março de 2012. Marcar as opções sim ou não para indicar se houve a ocorrência de cada um dos eventos abaixo descritos.
   - **Falha na aderência:** refere-se às situações nas quais a aderência da selagem está comprometida, deixando espaço livre entre as superfícies aderidas, de modo que o pacote não permanece fechado.
   - **Bolha:** pequena quantidade de ar contida numa superfície selada.
   - **Delaminação:** separação das camadas ou lâminas que compõem a embalagem.
   - **Queimadura:** marca deixada no papel decorrente de temperatura excessivamente alta.

- **Vinco (túnel):** refere-se à prega, sinuosidade, ondulação. O vinco pode favorecer a formação de túneis, comprometendo a integridade da selagem.

4. **INDICADOR QUÍMICO externo (fita zebrada e/ou área tingida na borda da embalagem):**
    - **Corado:** quando há presença do indicador químico na parte externa do pacote. Para o preenchimento do instrumento, considerar como indicador químico a fita zebrada, utilizada em embalagens SMS, e a área tingida na borda da embalagem de papel grau cirúrgico ou outro. Para avaliação quanto a coloração, seguir a indicação do fabricante.
    - **Não corado:** quando o indicador químico não atingiu a coloração esperada definida pelo fabricante após passar por esterilização.
    - **Ausente:** quando não há a presença do indicador químico na parte externa do pacote.

5. **INTERCORRÊNCIAS:** variação ou irregularidade; qualquer ocorrência que seja divergente do previsto. Para o preenchimento do instrumento, refere-se a ocorrência de uma ação que pode resultar, ou resultou, em dano à esterilidade do PPS. Se, em caso de intercorrências, o PPS for considerado pelo examinador como possivelmente "contaminado", este deverá retornar ao CME para ser avaliado. Marcar as opções sim ou não para indicar o desfecho da intercorrência.
    - **Suspeita de que o pacote tenha sido aberto:** presença de algum indício de que o pacote possa ter sido violado. Exemplo: resíduos de fita adesiva, delaminação, etc.
    - **Data limite de uso expirada:** conforme Art. 4º, item VII, da RDC nº 15, de 15 de março de 2012, data limite de uso refere-se ao prazo estabelecido em cada instituição, baseado em um plano de avaliação da integridade das embalagens.
    - **Pacote caiu no chão:** o pacote que cair no chão, independente da situação, deverá ser reavaliado imediatamente após a queda para verificação de prejuízos ou perda de integridade da embalagem. Para reavaliação do pacote, utilize o campo "reavaliação após queda" com as definições descritas no item 2 do instrumento, marcando todos os itens que estão presentes.

Profissional responsável pela conferência: identificação do profissional de saúde que fez a conferência da embalagem.
Data: data contendo dia, mês e ano da conferência do PPS.

Elaborado por: Vilas-Boas VA, Rondini LA, Czempik TCV, Lorenzetti AHM, Graziano KU, Dini AP. Construction and validation of an instrument for event-related sterility of processed healthcare products. Rev Bras Enferm. 2024;77(4):e20240021. https://doi.org/10.1590/0034-7167-2024-0021pt
