# Supplementary material for: Construction and validation of an instrument for event-related sterility of processed healthcare products
Source: Rev Bras Enferm. 2024 Sep 6;77(4):e20240021. doi: 10.1590/0034-7167-2024-0021 (PMC11382677; doi:10.1590/0034-7167-2024-0021)
Supplement: 0034-7167-reben-77-04-e20240021-suppl03 [file 0034-7167-reben-77-04-e20240021-suppl03.pdf]

		Nº ordem: ____	PREPARO	1. APRESENTAÇÃO DO PRODUTO	Embalagem:	simples	dupla	Proteção em caso de PPS perfurocortantes	sim	não
Juiz 1	Pertinência	0	1	1	1	0	0	1 1 1 1 1	1	1
Juiz 2	Pertinência	1	1	1	1	1	1		1	1
Juiz 3	Pertinência	1	1	1	1	1	1		1	1
Juiz 4	Pertinência	1	1	1	1	1	1		1	1
Juiz 5	Pertinência	1	1	1	1	0	0		1	1
	IVC	0.80	1.00	1.00	1.00	0.60	0.60	1.00	1.00	1.00
	Kappa modificado	0.76	1.00	1.00	1.00	0.42	0.42	1.00	1.00	1.00
Juiz 1	Clareza	1	1	1	1	1	1	1	1	1
Juiz 2	Clareza	1	1	1	1	1	1	1	1	1
Juiz 3	Clareza	0	1	1	1	1	1	0	1	1
Juiz 4	Clareza	1	1	1	1	1	1	1	1	1
Juiz 5	Clareza	1	1	1	1	1	1	1	1	1
	IVC	0.80	1.00	1.00	1.00	1.00	1.00	0.80	1.00	1.00
	Kappa modificado	0.76	1.00	1.00	1.00	1.00	1.00	0.76	1.00	1.00

	Abrangência dos domínios
Juiz 1	1
Juiz 2	1
Juiz 3	1
Juiz 4	1
Juiz 5	1
IVC	1.00
Kappa modificado	1.00

	Aparência geral do instrumento
Juiz 1	0
Juiz 2	1
Juiz 3	1
Juiz 4	1
Juiz 5	1

IVC	0.80
Kappa modificado	0.76

OBS: Para fins de cálculos estatísticos, as pontuações 1 e 2 dadas pelos juízes na escala tipo Likert foram consideradas 0 (zero), e as pontuções 3 e 4 foram consideradas 1 (um).

Etiqueta de identificação de preparo contendo: nome do produto, número de peças, data do preparo, presença de indicador, nome do preparador	sim	não	Etiqueta de identificação de esterilização contendo: nome do produto, número de peças, número do lote ou carga, data da esterilização, data limite de uso, método de esterilização, nome do responsável pela esterilização.	sim	não m	Marcações à caneta diretament e na embalage	sim	não	Rúbica/Carimbo:	Data	GUARDA E DISTRIBUIÇÃO
0	0	0	1	1	1	0	0	0	1	1	1
1	1	1	1	1	1	1	1	1	1	1	1
0	0	0	0	0	0	1	1	1	1	1	1
1	1	1	1	1	1	1	1	1	1	1	0
1	1	1	1	1	1	1	1	1	1	1	1
0.60	0.60	0.60	0.80	0.80	0.80	0.80	0.80	0.80	1.00	1.00	0.80
0.42	0.42	0.42	0.76	0.76	0.76	0.76	0.76	0.76	1.00	1.00	0.76
1	1	1	1	1	1	1	1	1	1	1	1
1	1	1	1	1	1	1	1	1	1	1	1
0	0	0	0	0	0	1	1	1	0	0	1
1	1	1	1	1	1	1	1	1	1	1	1
1	1	1	1	1	1	1	1	1	1	1	1
0.80	0.80	0.80	0.80	0.80	0.80	1.00	1.00	1.00	0.80	0.80	1.00
0.76	0.76	0.76	0.76	0.76	0.76	1.00	1.00	1.00	0.76	0.76	1.00

2. EVENTO RELACIONADO	O pacote a ser distribuído para as unidades apresenta:	Rasgo	sim	não	Corte	sim	não	Torção	sim	não	Furos/microfuros (olhar contra à luz em caso de papel grau cirúrgico)	sim	não	Manchas na embalagem ou PPS
1	0	1	1	1	1	1	1	0	1	1	1	1	1	1
1	1	1	1	1	1	1	1	1	1	1	1	1	1	1
1	1	1	1	1	1	1	1	1	1	1	1	1	1	1
0	0	0	0	0	0	0	0	0	0	0	1	1	1	1
1	1	1	1	1	1	1	1	1	1	1	1	1	1	1
0.80	0.60	0.80	0.80	0.80	0.80	0.80	0.80	0.60	0.80	0.80	1.00	1.00	1.00	1.00
0.76	0.42	0.76	0.76	0.76	0.76	0.76	0.76	0.42	0.76	0.76	1.00	1.00	1.00	1.00
1	1	1	1	1	1	1	1	0	1	1	1	1	1	0
1	1	1	1	1	1	1	1	1	1	1	1	1	1	1
1	1	1	1	1	1	1	1	1	1	1	1	1	1	1
1	1	1	1	1	1	1	1	1	1	1	1	1	1	1
1	1	1	1	1	1	1	1	1	1	1	1	1	1	1
1.00	1.00	1.00	1.00	1.00	1.00	1.00	1.00	0.80	1.00	1.00	1.00	1.00	1.00	0.80
1.00	1.00	1.00	1.00	1.00	1.00	1.00	1.00	0.76	1.00	1.00	1.00	1.00	1.00	0.76

sim	não	Umidade na embalagem ou PPS	sim	não	Sujidade na embalagem ou PPS	sim	não	3. SELAGEM DA EMBALAGEM	A selagem apresenta:	Falha na aderência	sim	não	3.1 Para PAPEL GRAU CIRÚRGICO
1	1	1	1	1	1	1	1	1	0	0	0	0	1
1	1	1	1	1	1	1	1	1	1	1	1	1	1
1	1	1	1	1	1	1	1	1	1	1	1	1	1
1	1	1	1	1	1	1	1	1	1	1	1	1	1
1	1	1	1	1	1	1	1	1	1	1	1	1	1
1.00	1.00	1.00	1.00	1.00	1.00	1.00	1.00	1.00	0.80	0.80	0.80	0.80	1.00
1.00	1.00	1.00	1.00	1.00	1.00	1.00	1.00	1.00	0.76	0.76	0.76	0.76	1.00
1	1	0	1	1	1	1	1	1	1	0	0	0	1
1	1	1	1	1	1	1	1	1	1	1	1	1	1
1	1	1	1	1	1	1	1	1	1	1	1	1	1
1	1	1	1	1	0	0	0	1	1	1	1	1	1
1	1	1	1	1	1	1	1	1	1	1	1	1	1
1.00	1.00	0.80	1.00	1.00	0.80	0.80	0.80	1.00	1.00	0.80	0.80	0.80	1.00
1.00	1.00	0.76	1.00	1.00	0.76	0.76	0.76	1.00	1.00	0.76	0.76	0.76	1.00

A selagem apresenta: Bolha		sim	não	Delaminação	sim	não	Queimadura	sim	não	Dobra ou Vinco	sim	não	4. INDICADOR QUÍMICO
1	1	1	1	1	1	1	0	0	0	1	1	1	1
1	1	1	1	1	1	1	1	1	1	1	1	1	1
1	1	1	1	1	1	1	1	1	1	1	1	1	1
1	1	1	1	1	1	1	1	1	1	1	1	1	1
1	1	1	1	1	1	1	1	1	1	1	1	1	1
1.00	1.00	1.00	1.00	1.00	1.00	1.00	0.80	0.80	0.80	1.00	1.00	1.00	1.00
1.00	1.00	1.00	1.00	1.00	1.00	1.00	0.76	0.76	0.76	1.00	1.00	1.00	1.00
1	1	1	1	1	1	1	1	1	1	1	1	1	1
1	1	1	1	1	1	1	1	1	1	1	1	1	1
1	1	1	1	1	1	1	1	1	1	1	1	1	0
1	1	1	1	1	1	1	1	1	1	1	1	1	1
1	1	1	1	1	1	1	1	1	1	1	1	1	1
1.00	1.00	1.00	1.00	1.00	1.00	1.00	1.00	1.00	1.00	1.00	1.00	1.00	0.80
1.00	1.00	1.00	1.00	1.00	1.00	1.00	1.00	1.00	1.00	1.00	1.00	1.00	0.76

Corado	Ausente	Falha na coloração	Rúbrica/Carimbo:	Data	A QUALQUER MOMENTO	5. INTERCORRÊNCIAS	Suspeita de que o pacote tenha sido aberto	sim	não	Data limite de uso expirada	sim	não
1	1	1	1	1	1	1	1	1	1	1	1	1
1	1	1	1	1	1	1	1	1	1	1	1	1
1	1	1	1	1	1	1	1	1	1	1	1	1
1	1	1	1	1	1	1	1	1	1	1	1	1
1	1	1	0	1	1	1	1	1	1	1	1	1
1.00	1.00	1.00	0.80	1.00	1.00	1.00	1.00	1.00	1.00	1.00	1.00	1.00
1.00	1.00	1.00	0.76	1.00	1.00	1.00	1.00	1.00	1.00	1.00	1.00	1.00
1	1	1	1	1	1	1	1	1	1	1	1	1
1	1	1	1	1	1	1	1	1	1	1	1	1
0	0	0	1	1	0	1	1	1	1	1	1	1
1	1	1	1	1	1	1	1	1	1	1	1	1
1	1	1	0	1	1	1	1	1	1	1	1	1
0.80	0.80	0.80	0.80	1.00	0.80	1.00	1.00	1.00	1.00	1.00	1.00	1.00
0.76	0.76	0.76	0.76	1.00	0.76	1.00	1.00	1.00	1.00	1.00	1.00	1.00

Pacote caiu no chão	sim*	não	*REAVALIAÇÃO APÓS QUEDA*	Rasgo	Corte	Torção	Furos/microfuros	Manchas	Umidade	Sujidade	Não se aplica	Rúbica/Carimbo: Data	
1	1	1	1	1	1	1	1	1	1	1	1	1	1
1	1	1	1	1	1	1	1	1	1	1	1	1	1
1	1	1	1	1	1	1	1	1	1	1	1	1	1
1	1	1	1	1	1	1	1	1	1	1	1	1	1
1	1	1	1	1	1	1	1	1	1	1	1	0	1
1.00	1.00	1.00	1.00	1.00	1.00	1.00	1.00	1.00	1.00	1.00	1.00	0.80	1.00
1.00	1.00	1.00	1.00	1.00	1.00	1.00	1.00	1.00	1.00	1.00	1.00	0.76	1.00
1	1	1	1	1	1	1	1	1	1	1	1	1	1
1	1	1	1	1	1	1	1	1	1	1	1	1	1
0	0	0	1	1	1	1	1	1	1	1	0	0	0
1	1	1	1	0	0	0	1	1	0	1	1	1	1
1	1	1	1	1	1	1	1	1	1	1	1	0	1
0.80	0.80	0.80	1.00	0.80	0.80	0.80	1.00	1.00	0.80	1.00	0.80	0.60	0.80
0.76	0.76	0.76	1.00	0.76	0.76	0.76	1.00	1.00	0.76	1.00	0.76	0.42	0.76

		1. APRESENTAÇÃO DO PRODUTO	2. EVENTO RELACIONADO	3. SELAGEM DA EMBALAGEM	4. INDICADOR QUÍMICO	5. INTERCORRÊNCIAS	*Reavaliação após queda	Rúbrica/Carimbo	Data
Juiz 1	Pertinência	1	1	1	1	1	1	1	1
Juiz 2	Pertinência	1	1	1	1	1	1	1	1
Juiz 3	Pertinência	1	1	1	1	1	1	1	1
Juiz 4	Pertinência	1	1	1	1	1	1	1	1
Juiz 5	Pertinência	1	1	1	1	1	1	0	1
	IVC	1.00	1.00	1.00	1.00	1.00	1.00	0.80	1.00
	Kappa modificado	1.00	1.00	1.00	1.00	1.00	1.00	0.76	1.00
Juiz 1	Clareza	1	1	1	1	1	1	1	1
Juiz 2	Clareza	1	1	1	1	1	1	1	1
Juiz 3	Clareza	0	1	1	1	1	1	1	1
Juiz 4	Clareza	1	1	1	1	1	0	1	1
Juiz 5	Clareza	1	1	1	1	1	1	0	1
	IVC	0.80	1.00	1.00	1.00	1.00	0.80	0.80	1.00
	Kappa modificado	0.76	1.00	1.00	1.00	1.00	0.76	0.76	1.00
Juiz 1	Abrangência	1	1	0	1	1	1	1	1
Juiz 2	Abrangência	1	1	1	1	1	1	1	1
Juiz 3	Abrangência	0	1	1	1	1	1	1	1
Juiz 4	Abrangência	1	1	1	1	1	1	1	1
Juiz 5	Abrangência	1	1	1	1	1	1	0	1
	IVC	0.80	1.00	0.80	1.00	1.00	1.00	0.80	1.00
	Kappa modificado	0.76	1.00	0.76	1.00	1.00	1.00	0.76	1.00

	Aparência geral do guia
Juiz 1	0
Juiz 2	1
Juiz 3	1
Juiz 4	1
Juiz 5	1
IVC	0.80
Kappa modificado	0.76

		Nº ordem: ____	Identificação do produto:	1. APRESENTAÇÃO DO PRODUTO	O pacote contém:	Proteção em caso de PPS perfurocortantes	sim	não
Juiz 1	Pertinência	1	1	1	1	1	1	1
Juiz 2	Pertinência	1	1	1	1	1	1	1
Juiz 3	Pertinência	1	1	1	1	1	1	1
Juiz 4	Pertinência	1	1	1	1	1	1	1
Juiz 6	Pertinência	1	1	1	1	1	1	1
	IVC	1.00	1.00	1.00	1.00	1.00	1.00	1.00
	Kappa modificado	1.00	1.00	1.00	1.00	1.00	1.00	1.00

Juiz 1	Clareza	1	1	1	1	1	1	1
Juiz 2	Clareza	1	1	1	1	1	1	1
Juiz 3	Clareza	1	0	1	1	1	1	1
Juiz 4	Clareza	1	1	1	1	1	1	1
Juiz 6	Clareza	1	1	1	1	1	1	1
	IVC	1.00	0.80	1.00	1.00	1.00	1.00	1.00
	Kappa modificado	1.00	0.76	1.00	1.00	1.00	1.00	1.00

	Abrangência dos domínios
Juiz 1	0
Juiz 2	1
Juiz 3	1
Juiz 4	1
Juiz 6	1
IVC	0.80
Kappa modificado	0.76

não se aplica	Etiqueta de identificação de esterilização contendo: nome do produto, número de peças, número do lote ou carga, data da esterilização, data limite de uso, método de esterilização, nome do responsável pela esterilização.	sim	não	2. EVENTO RELACIONADO	O pacote apresenta:	Rasgo/corte	sim
1	1	1	1	1	1	1	1
1	1	1	1	1	1	1	1
1	1	1	1	1	1	1	1
1	1	1	1	1	1	1	1
1	1	1	1	1	1	1	1
1.00	1.00	1.00	1.00	1.00	1.00	1.00	1.00
1.00	1.00	1.00	1.00	1.00	1.00	1.00	1.00
1	1	1	1	1	1	1	1
1	1	1	1	1	1	1	1
1	1	1	1	1	1	1	1
1	1	1	1	1	1	1	1
1	1	1	1	1	1	1	1
1.00	1.00	1.00	1.00	1.00	1.00	1.00	1.00
1.00	1.00	1.00	1.00	1.00	1.00	1.00	1.00

não	Dobra	sim	não	Furos/micro furos (olhar contra à luz em caso de papel grau cirúrgico)	sim	não	Marcações à caneta diretamente na embalagem	sim
1	1	1	1	1	1	1	1	1
1	1	1	1	1	1	1	1	1
1	1	1	1	1	1	1	1	1
1	1	1	1	1	1	1	1	1
1	1	1	1	1	1	1	1	1
1.00	1.00	1.00	1.00	1.00	1.00	1.00	1.00	1.00
1.00	1.00	1.00	1.00	1.00	1.00	1.00	1.00	1.00
1	1	1	1	1	1	1	1	1
1	1	1	1	1	1	1	1	1
1	1	1	1	1	1	1	1	1
1	1	1	1	1	1	1	1	1
1	1	1	1	1	1	1	1	1
1.00	1.00	1.00	1.00	1.00	1.00	1.00	1.00	1.00
1.00	1.00	1.00	1.00	1.00	1.00	1.00	1.00	1.00

não	Umidade na embalagem ou PPS	sim	não	Sujidade/mancha na embalagem ou PPS	sim	não	3. SELAGEM DA EMBALAGEM	A selagem apresenta:
1	1	1	1	1	1	1	1	1
1	1	1	1	1	1	1	1	1
1	1	1	1	1	1	1	1	1
1	1	1	1	1	1	1	1	1
1	1	1	1	1	1	1	1	1
1.00	1.00	1.00	1.00	1.00	1.00	1.00	1.00	1.00
1.00	1.00	1.00	1.00	1.00	1.00	1.00	1.00	1.00
1	1	1	1	1	1	1	0	1
1	1	1	1	1	1	1	1	1
1	1	1	1	1	1	1	1	1
1	1	1	1	1	1	1	1	1
1	1	1	1	1	1	1	1	1
1.00	1.00	1.00	1.00	1.00	1.00	1.00	0.80	1.00
1.00	1.00	1.00	1.00	1.00	1.00	1.00	0.76	1.00

Falha na aderência	sim	não	Bolha	sim	não	Delaminação	sim	não
1	1	1	1	1	1	1	1	1
1	1	1	1	1	1	1	1	1
1	1	1	1	1	1	1	1	1
1	1	1	1	1	1	1	1	1
1	1	1	1	1	1	1	1	1
1.00	1.00	1.00	1.00	1.00	1.00	1.00	1.00	1.00
1.00	1.00	1.00	1.00	1.00	1.00	1.00	1.00	1.00
1	1	1	1	1	1	1	1	1
1	1	1	1	1	1	1	1	1
1	1	1	1	1	1	1	1	1
1	1	1	1	1	1	1	1	1
1	1	1	1	1	1	1	1	1
1.00	1.00	1.00	1.00	1.00	1.00	1.00	1.00	1.00
1.00	1.00	1.00	1.00	1.00	1.00	1.00	1.00	1.00

Queimadura	sim	não	Vinco (túnel)	sim	não	4. INDICADOR QUÍMICO EXTERNO (fita zebrada e/ou área tingida na borda da embalagem)	Corado	Não corado
˥ ˥ ˥ ˥ ˥	˥ ˥ ˥ ˥ ˥	˥ ˥ ˥ ˥ ˥	˥ ˥ ˥ ˥ ˥	˥ ˥ ˥ ˥ ˥	˥ ˥ ˥ ˥ ˥	˥ ˥ ˥ ˥ ˥	˥ ˥ ˥ ˥ ˥	˥ ˥ ˥ ˥ ˥
1.00	1.00	1.00	1.00	1.00	1.00	1.00	1.00	1.00
1.00	1.00	1.00	1.00	1.00	1.00	1.00	1.00	1.00
˥ ˥ ˥ ˥ ˥	˥ ˥ ˥ ˥ ˥	˥ ˥ ˥ ˥ ˥	˥ ˥ ˥ ˥ ˥	˥ ˥ ˥ ˥ ˥	˥ ˥ ˥ ˥ ˥	˥ ˥ ˥ ˥ ˥	˥ ˥ ˥ ˥ ˥	˥ ˥ ˥ ˥ ˥
1.00	1.00	1.00	1.00	1.00	1.00	1.00	1.00	1.00
1.00	1.00	1.00	1.00	1.00	1.00	1.00	1.00	1.00

Ausente	5. INTERCORRÊNCIAS	Suspeita de que o pacote tenha sido aberto	sim	não	Data limite de uso expirada	sim	não	Pacote caiu no chão
1	1	1	1	1	1	1	1	0
1	1	1	1	1	1	1	1	1
1	1	1	1	1	1	1	1	1
1	1	1	1	1	1	1	1	1
1	1	1	1	1	1	1	1	1
1.00	1.00	1.00	1.00	1.00	1.00	1.00	1.00	0.80
1.00	1.00	1.00	1.00	1.00	1.00	1.00	1.00	0.76
1	1	1	1	1	1	1	1	0
1	1	1	1	1	1	1	1	1
1	1	1	1	1	1	1	1	1
1	1	1	1	1	1	1	1	1
1	1	1	1	1	1	1	1	1
1.00	1.00	1.00	1.00	1.00	1.00	1.00	1.00	0.80
1.00	1.00	1.00	1.00	1.00	1.00	1.00	1.00	0.76

sim*	não	*REAVALIAÇÃO APÓS QUEDA*	Rasgo/corte	Sujidade	Manchas	Dobra	Umidade	Furos/micro furos
˥	˥	˥	˥	˥	˥	˥	˥	˥
˥	˥	˥	˥	˥	˥	˥	˥	˥
˥	˥	˥	˥	˥	˥	˥	˥	˥
˥	˥	˥	˥	˥	˥	˥	˥	˥
˥	˥	˥	˥	˥	˥	˥	˥	˥
1.00	1.00	1.00	1.00	1.00	1.00	1.00	1.00	1.00
1.00	1.00	1.00	1.00	1.00	1.00	1.00	1.00	1.00
˥	˥	˥	˥	˥	˥	˥	˥	˥
˥	˥	˥	˥	˥	˥	˥	˥	˥
˥	˥	˥	˥	˥	˥	˥	˥	˥
˥	˥	˥	˥	˥	˥	˥	˥	˥
˥	˥	˥	˥	˥	˥	˥	˥	˥
1.00	1.00	1.00	1.00	1.00	1.00	1.00	1.00	1.00
1.00	1.00	1.00	1.00	1.00	1.00	1.00	1.00	1.00

Não se aplica	Avalie os itens 2 a 5, se identificar qualquer não conformidade de NÃO utilizar o pacote. Devolvê-lo ao CME para ser avaliado.	Profissional responsável pela conferência	Data
1	1	1	1
1	1	1	1
1	1	1	1
1	1	1	1
1	1	1	1
1.00	1.00	1.00	1.00
1.00	1.00	1.00	1.00
1	1	1	1
1	1	1	1
1	1	1	1
1	1	1	1
1	1	1	1
1.00	1.00	1.00	1.00
1.00	1.00	1.00	1.00

		1. APRESENTAÇÃO DO PRODUTO	2. EVENTO RELACIONA DO	3. SELAGEM DA EMBALAGEM	4. INDICADOR QUÍMICO	5. INTERCORRÊNCIAS	*Reavaliação após queda	Profissional responsável pela conferência	Data
Juiz 1	Pertinência	1	1	1	1	1	1	1	1
Juiz 2	Pertinência	1	1	1	1	1	1	1	1
Juiz 3	Pertinência	1	1	1	1	1	1	1	1
Juiz 4	Pertinência	1	1	1	1	1	1	1	1
Juiz 6	Pertinência	1	1	1	1	1	1	1	1
	IVC	1.00	1.00	1.00	1.00	1.00	1.00	1.00	1.00
	Kappa modificado	1.00	1.00	1.00	1.00	1.00	1.00	1.00	1.00
Juiz 1	Clareza	1	1	1	1	1	0	1	1
Juiz 2	Clareza	1	1	1	1	1	1	1	1
Juiz 3	Clareza	1	1	0	1	1	0	1	1
Juiz 4	Clareza	1	1	1	1	1	1	1	1
Juiz 6	Clareza	1	1	1	1	1	1	1	1
	IVC	1.00	1.00	0.80	1.00	1.00	0.60	1.00	1.00
	Kappa modificado	1.00	1.00	0.76	1.00	1.00	0.42	1.00	1.00
Juiz 1	Abrangência	1	1	1	1	1	1	1	1
Juiz 2	Abrangência	1	1	1	1	1	1	1	1
Juiz 3	Abrangência	1	1	1	1	1	0	1	1
Juiz 4	Abrangência	1	1	1	1	1	1	1	1
Juiz 6	Abrangência	1	1	1	1	1	1	1	1
	IVC	1.00	1.00	1.00	1.00	1.00	0.80	1.00	1.00
	Kappa modificado	1.00	1.00	1.00	1.00	1.00	0.76	1.00	1.00
