# Supplementary material for: Construction and validation of an instrument for event-related sterility of processed healthcare products
Source: Rev Bras Enferm. 2024 Sep 6;77(4):e20240021. doi: 10.1590/0034-7167-2024-0021 (PMC11382677; doi:10.1590/0034-7167-2024-0021)
Supplement: 0034-7167-reben-77-04-e20240021-suppl04 [file 0034-7167-reben-77-04-e20240021-suppl04.pdf]

AVALIAÇÃO DE EVENTO RELACIONADO À MANUTENÇÃO DA ESTERILIDADE DE PRODUTOS PARA SAÚDE PROCESSADOS (AERMS)

Quantidade de avaliações do produto:________

Identificação do produto: ___________

1.APRESENTAÇÃO DO PRODUTO

O pacote contém:

Proteção em caso de PPS perfuro cortantes:

☐ sim ☐ não ☐ não se aplica

Etiqueta de identificação da **esterilização** contendo: nome do produto, número de peças, número do lote ou carga, data da esterilização, data limite de uso, método de esterilização, nome do responsável pela esterilização: ☐ sim ☐ não

2.EVENTO RELACIONADO

O pacote apresenta:

Rasgo/corte: ☐ sim ☐ não

Dobra: ☐ sim ☐ não

Furos/microfuros (olhar contra a luz para grau cirúrgico): ☐ sim ☐ não

Marcações à caneta diretamente na embalagem:

☐ sim ☐ não

Umidade na embalagem ou PPS: ☐ sim ☐ não

Sujidade/mancha na embalagem ou PPS:

☐ sim ☐ não

3.SELAGEM DA EMBALAGEM

A selagem apresenta:

Falha na aderência: ☐ sim ☐ não

Bolha: ☐ sim ☐ não

Delaminação: ☐ sim ☐ não

Queimadura: ☐ sim ☐ não

Vinco (túnel): ☐ sim ☐ não

4.INDICADOR QUÍMICO EXTERNO (fita zebrada e/ou área tingida na borda da embalagem)

☐ corado ☐ não corado ☐ ausente

5.INTERCORRÊNCIAS

Suspeita de que o pacote tenha sido aberto:

☐ sim ☐ não

Data limite de uso expirada: ☐ sim ☐ não

Pacote caiu no chão: ☐ sim* ☐ não

***Se sim, fazer a REAVALIAÇÃO APÓS QUEDA:**

☐ rasgo/corte ☐ sujidade ☐ manchas

☐ dobra ☐ umidade ☐ furos/microfuros

☐ não houve dano à integridade da embalagem

Avalie os itens 2 a 5, se identificar qualquer não conformidade **NÃO** utilizar o pacote. Devolvê-lo ao CME para ser avaliado.

Profissional responsável pela conferência:

____

Data: ____/_____/_____

AVALIAÇÃO DE EVENTO RELACIONADO À MANUTENÇÃO DA ESTERILIDADE DE PRODUTOS PARA SAÚDE PROCESSADOS (AERMS)

Quantidade de avaliações do produto:________

Identificação do produto: ___________

1.APRESENTAÇÃO DO PRODUTO

O pacote contém:

Proteção em caso de PPS perfuro cortantes:

☐ sim ☐ não ☐ não se aplica

Etiqueta de identificação da esterilização contendo: nome do produto, número de peças, número do lote ou carga, data da esterilização, data limite de uso, método de esterilização, nome do responsável pela esterilização: ☐ sim ☐ não

2.EVENTO RELACIONADO

O pacote apresenta:

Rasgo/corte: ☐ sim ☐ não

Dobra: ☐ sim ☐ não

Furos/microfuros (olhar contra a luz para grau cirúrgico): ☐ sim ☐ não

Marcações à caneta diretamente na embalagem:

☐ sim ☐ não

Umidade na embalagem ou PPS: sim não

Sujidade/mancha na embalagem ou PPS:

☐ sim ☐ não

3.SELAGEM DA EMBALAGEM

A selagem apresenta:

Falha na aderência: ☐ sim ☐ não

Bolha: ☐ sim ☐ não

Delaminação: ☐ sim ☐ não

Queimadura: ☐ sim ☐ não

Vinco (túnel): ☐ sim ☐ não

4.INDICADOR QUÍMICO EXTERNO (fita zebrada e/ou área tingida na borda da embalagem)

☐ corado ☐ não corado ☐ ausente

5.INTERCORRÊNCIAS

Suspeita de que o pacote tenha sido aberto:

☐ sim ☐ não

Data limite de uso expirada: ☐ sim ☐ não

Pacote caiu no chão: ☐ sim* ☐ não

*Se sim, fazer a REAVALIAÇÃO APÓS QUEDA:

☐ rasgo/corte ☐ sujidade ☐ manchas

☐ dobra ☐ umidade ☐ furos/microfuros

☐ não houve dano à integridade da embalagem

Avalie os itens 2 a 5, se identificar qualquer não conformidade NÃO utilizar o pacote. Devolvê-lo ao CME para ser avaliado.

Profissional responsável pela conferência:

____

Data: ____/_____/_____

GUIA DE INSTRUÇÕES PARA PREENCHIMENTO DO INSTRUMENTO (RESUMIDO)

Quantidade de avaliações do produto:: nº fornecido ao impresso no momento da avaliação da embalagem.
Identificação do produto: nome do produto.
Proteção em caso de PPS perfurocortantes: os PPS perfurocortantes, acondicionados em papel grau cirúrgico, deverão ter um dispositivo de proteção para proteger a embalagem principal de perfurações e rasgos.
Etiqueta de identificação da *esterilização* contendo: nome do produto, número de peças, número do lote ou carga, data da esterilização, data limite de uso, método de esterilização, nome do responsável pela esterilização: etiqueta autocolante fixada na parte externa da embalagem.
Rasgo/corte: abertura numa superfície que se rompeu ou dilacerou; separação ou divisão por meio de instrumento cortante.
Dobra: Dobrar, inclinar, vergar, encurvar. Nesse caso, avaliar se houve ruptura, fissura e/ou separação da superfície da embalagem que prejudique a esterilidade do produto.
Furos/microfuros: perfurações, orifício, buraco. Para grau cirúrgico, é válida a visualização contra a luz.
Marcações à caneta diretamente na embalagem: A marcação à caneta ou outro tipo de tinta diretamente na embalagem proporciona risco de dano à embalagem e transferência de tinta para o PPS.
Umidade na embalagem ou PPS: característica do que está úmido, envolto por vapor de água, sutilmente molhado.
Sujidade/mancha na embalagem ou PPS: Marca de cor diferente presente na superfície da embalagem ou do PPS. Refere-se à qualidade do que é sujo, imundície, sujeira.
Falha na aderência: quando a aderência da selagem está comprometida.
Bolha: pequena quantidade de ar contida numa superfície selada..
Delaminação: separação das camadas ou lâminas que compõem a embalagem.
Queimadura: marca deixada no papel decorrente de temperatura excessivamente alta.
Vinco (túnel): prega, sinuosidade, ondulação. Pode favorecer a formação de túneis.
Corado: quando há presença do indicador químico na parte externa do pacote. Para avaliação quanto a coloração, seguir a indicação do fabricante.
Não corado: quando o indicador químico não atingiu a coloração esperada definida pelo fabricante.
Ausente: quando não há a presença do indicador.
Suspeita de que o pacote tenha sido aberto: presença de algum indício de que o pacote possa ter sido violado.
Data limite de uso expirada: refere-se ao prazo de validade estabelecido em cada instituição.
Pacote caiu no chão: o pacote que cair no chão deverá ser reavaliado imediatamente após a queda para verificação de prejuízos ou perda de integridade da embalagem. Caso marcado "sim*", preencher item "REAVALIAÇÃO APÓS QUEDA".

Guia completo disponível na unidade

GUIA DE INSTRUÇÕES PARA PREENCHIMENTO DO INSTRUMENTO (RESUMIDO)

Quantidade de avaliações do produto:: nº fornecido ao impresso no momento da avaliação da embalagem.
Identificação do produto: nome do produto.
Proteção em caso de PPS perfurocortantes: os PPS perfurocortantes, acondicionados em papel grau cirúrgico, deverão ter um dispositivo de proteção para proteger a embalagem principal de perfurações e rasgos.
Etiqueta de identificação da *esterilização* contendo: nome do produto, número de peças, número do lote ou carga, data da esterilização, data limite de uso, método de esterilização, nome do responsável pela esterilização: etiqueta autocolante fixada na parte externa da embalagem.
Rasgo/corte: abertura numa superfície que se rompeu ou dilacerou; separação ou divisão por meio de instrumento cortante.
Dobra: Dobrar, inclinar, vergar, encurvar. Nesse caso, avaliar se houve ruptura, fissura e/ou separação da superfície da embalagem que prejudique a esterilidade do produto.
Furos/microfuros: perfurações, orifício, buraco. Para grau cirúrgico, é válida a visualização contra a luz.
Marcações à caneta diretamente na embalagem: A marcação à caneta ou outro tipo de tinta diretamente na embalagem proporciona risco de dano à embalagem e transferência de tinta para o PPS.
Umidade na embalagem ou PPS: característica do que está úmido, envolto por vapor de água, sutilmente molhado.
Sujidade/mancha na embalagem ou PPS: Marca de cor diferente presente na superfície da embalagem ou do PPS. Refere-se à qualidade do que é sujo, imundície, sujeira.
Falha na aderência: quando a aderência da selagem está comprometida.
Bolha: pequena quantidade de ar contida numa superfície selada..
Delaminação: separação das camadas ou lâminas que compõem a embalagem.
Queimadura: marca deixada no papel decorrente de temperatura excessivamente alta.
Vinco (túnel): prega, sinuosidade, ondulação. Pode favorecer a formação de túneis.
Corado: quando há presença do indicador químico na parte externa do pacote. Para avaliação quanto a coloração, seguir a indicação do fabricante.
Não corado: quando o indicador químico não atingiu a coloração esperada definida pelo fabricante.
Ausente: quando não há a presença do indicador.
Suspeita de que o pacote tenha sido aberto: presença de algum indício de que o pacote possa ter sido violado.
Data limite de uso expirada: refere-se ao prazo de validade estabelecido em cada instituição.
Pacote caiu no chão: o pacote que cair no chão deverá ser reavaliado imediatamente após a queda para verificação de prejuízos ou perda de integridade da embalagem. Caso marcado "sim*", preencher item "REAVALIAÇÃO APÓS QUEDA".

Guia completo disponível na unidade
