# Supplementary material for: Construction and validation of an instrument for event-related sterility of processed healthcare products
Source: Rev Bras Enferm. 2024 Sep 6;77(4):e20240021. doi: 10.1590/0034-7167-2024-0021 (PMC11382677; doi:10.1590/0034-7167-2024-0021)
Supplement: 0034-7167-reben-77-04-e20240021-suppl05 [file 0034-7167-reben-77-04-e20240021-suppl05.pdf]

AVALIAÇÃO DE PRODUTOS PARA SAÚDE (PPS) ESTERILIZADOS   Nº ordem: _________

PREPARO

1.APRESENTAÇÃO DO PRODUTO

Embalagem: ☐ simples ☐ dupla

Proteção em caso de PPS perfuro cortantes:

☐ sim ☐ não

Etiqueta de identificação do **preparo** contendo: nome do produto, número de peças, data do preparo, presença de indicador, nome do preparador:

☐ sim ☐ não

Etiqueta de identificação da **esterilização** contendo: nome do produto, número de peças, número do lote ou carga, data da esterilização, data limite de uso, método de esterilização, nome do responsável pela esterilização:

☐ sim ☐ não

Marcações à caneta diretamente na embalagem:

☐ sim ☐ não

Rúbrica/Carimbo:__________________

Data:__/__/_____

GUARDA E DISTRIBUIÇÃO

2. EVENTO RELACIONADO

O pacote a ser distribuído para as unidades apresenta:

Rasgo: ☐ sim ☐ não   Corte: ☐ sim ☐ não

Torção: ☐ sim ☐ não

Furos/microfuros (olhar contra a luz para grau cirúrgico): ☐ sim ☐ não

Manchas na embalagem ou PPS: ☐ sim ☐ não

Umidade na embalagem ou PPS: ☐ sim ☐ não

Sujidade na embalagem ou PPS: ☐ sim ☐ não

3.SELAGEM DA EMBALAGEM

A selagem apresenta:

Falha na aderência: ☐ sim ☐ não

3.1 Para PAPEL GRAU CIRÚRGICO

A selagem apresenta:

Bolha: ☐ sim ☐ não

Delaminação: ☐ sim ☐ não

Queimadura: ☐ sim ☐ não

Dobra ou vinco: ☐ sim ☐ não

4.INDICADOR QUÍMICO

☐ corado ☐ ausente

☐ falha na coloração

Rúbrica/Carimbo:__________________

Data:__/__/_____

A QUALQUER MOMENTO

5.INTERCORRÊNCIAS

Suspeita de que o pacote tenha sido aberto:

☐ sim ☐ não

Data limite de uso expirada: ☐ sim ☐ não

Pacote caiu no chão: ☐ sim* ☐ não

***REAVALIAÇÃO APÓS QUEDA*:**

☐ rasgo	☐ manchas
☐ corte	☐ umidade
☐ torção	☐ sujidade
☐ furos/microfuros	☐ não se aplica

Rúbrica/Carimbo:__________________

Data:__/__/_____
