# Supplementary material for: Construction and validation of an instrument for event-related sterility of processed healthcare products
Source: Rev Bras Enferm. 2024 Sep 6;77(4):e20240021. doi: 10.1590/0034-7167-2024-0021 (PMC11382677; doi:10.1590/0034-7167-2024-0021)
Supplement: Translation

# Translation: Construção e validação de instrumento para esterilidade relacionada a eventos de produtos para saúde processados

**DOI:** 10.1590/0034-7167-2024-0021pt

**Authors:** Vanessa Aparecida Vilas-Boas, Louise Assumpção Rondini, Thamiris Cavazzani Vegro Czempik, Ada Helena Melo Lorenzetti, Kazuko Uchikawa Graziano, Ariane Polidoro Dini

**Affiliations:** I Universidade Estadual de Campinas. Campinas, São Paulo, Brasil; II Universidade Estadual de Campinas, Hospital da Mulher Prof. Dr. José Aristodemo Pinotti. Campinas, São Paulo, Brasil; III Universidade de São Paulo. São Paulo, São Paulo, Brasil

**Keywords:** Esterilização, Prazo de Validade de Produtos, Estudo de Validação, Avaliação em Enfermagem, Armazenamento de Produtos

## Abstract

**Objetivo:**

Construir e validar instrumento para avaliar evento relacionado à manutenção da esterilidade de produtos para saúde processados.

**Métodos:**

Estudo metodológico para desenvolver instrumento mediante análise por comitê de juízes. Considerou-se como aspecto norteador a integridade das embalagens mais utilizadas na prática: a de não tecido e a de papel grau cirúrgico. A análise foi realizada pelo Índice de Validade de Conteúdo, Razão de Validade de Conteúdo ≥ 0,80 e Kappa modificado ≥ 0,74. Submeteu-se o instrumento ao pré-teste.

**Resultados:**

Participaram do comitê de juízes seis profissionais experientes na temática. Após duas rodadas, chegou-se à versão final do instrumento, contendo cinco dimensões. No pré-teste, participaram 30 profissionais de enfermagem, dos quais 86,67% consideraram o instrumento bom; e 90%, compreensível.

**Conclusões:**

A construção e validação seguiu as recomendações da literatura. O instrumento está disponível para ser utilizado, auxiliando no uso seguro do produto para saúde.

## INTRODUÇÃO

As infecções relacionadas à assistência à saúde afetam milhões de pessoas mundialmente, constituindo um problema de saúde pública. Em particular, a infecção de sítio cirúrgico é comum, sendo considerada um evento adverso evitável. Recentemente, a Organização Mundial da Saúde propôs sete objetivos estratégicos para serem alcançados até 2030. Dentre eles, o Objetivo 3 defende garantir a segurança de todos os processos clínicos, os quais envolvem ações de implementação de medidas rigorosas para prevenção e controle de infecções e de segurança no uso de dispositivos médicos^(1)^.

Eventos relacionados à manutenção da esterilidade de produtos para saúde (PPS) são aqueles que podem causar dano à integridade da embalagem ou selagem do produto, decorrente de condições ambientais ou comportamentais adversas de armazenamento^(2)^ Tais danos podem ser prejudiciais, uma vez que predispõem o PPS que será utilizado no paciente à contaminação. Assim, o controle da esterilidade deve ser aprimorado por análise dos eventos ocorridos com o PPS, visto que sua contaminação pode ocorrer a qualquer momento, inclusive imediatamente após o processamento^(2, 3, 4)^, Entretanto, ainda muitas instituições optam por uma data arbitrária para o prazo de validade de esterilização, sem uma avaliação rigorosa e sistemática da ocorrência desses eventos.

O prazo de validade de esterilização de PPS processados pelo Centro de Material e Esterilização (CME) baseou-se historicamente em estudos da década de 1970, com aplicação de testes microbiológicos em produtos esterilizados sob condições adversas de armazenamento. Os autores consideraram que houve contaminação a partir do terceiro dia após esterilização para produtos embalados em tecido simples e 28 dias em tecido dupla camada em prateleiras abertas, com manutenção da esterilidade por 63 dias ou mais quando utilizado saco protetor^(5)^. Somente a partir de 1984, houve a ponderação de que o prazo de validade em estudos anteriores baseou-se no tempo, e não no controle de outras variáveis, como temperatura, umidade, local, transporte, movimentação do ar e embalagem, que são propriedades capazes de contaminar um material^(6)^. Surge, portanto, um conceito sobre a esterilidade relacionada a eventos, que se contrapõe ao paradigma da esterilidade relacionada ao tempo.

Ao longo dos anos, tem sido evidente que o tempo de armazenamento de PPS, desde que dentro das recomendações, não tem efeito sobre a susceptibilidade de contaminação desse material, mesmo que deliberadamente exposto a microrganismos^(3, 7)^. No século XIX, Louis Pasteur comprovou que, em amostras estéreis, só existe a proliferação de microrganismos por meio do contato destes com local já contaminado^(8)^.

Por essa lógica, produtos esterilizados mantidos com embalagem e selagem íntegras permanecem estéreis até que ocorra um evento ou dano à integridade do pacote. Isso porque sistemas de embalagem de esterilização devem promover segurança por meio das seguintes características: barreira à entrada de microrganismos, selagem hermética, proteção do conteúdo do pacote contra danos físicos e resistência às punções e rasgos^(7)^. Como exemplo, embalagens de papel grau cirúrgico e não tecido de spunbond-meltblown-spunbond (SMS) têm sido amplamente utilizadas para esterilização de PPS por apresentar baixo custo, ótima barreira antimicrobiana, alta permeabilidade aos esterilizantes e boa resistência mecânica^(7)^.

O CME é a unidade responsável pelo processamento de PPS, exercendo as seguintes funções: limpeza, inspeção, empacotamento, esterilização e distribuição às unidades assistenciais^(9)^. Porém, ainda que todas as etapas conduzidas dentro do CME atendam aos padrões de qualidade e segurança, a esterilidade do processamento poderá ser comprometida caso as unidades assistenciais não dispensem cuidado no manuseio, transporte e armazenamento e não se atentem à qualidade da selagem e embalagem dos PPS processados^(2, 10)^.

Em um estudo brasileiro feito com 11 hospitais de grande porte, foi evidente o desconhecimento dos profissionais de enfermagem sobre as etapas do processamento do PPS, tipos de embalagens adotadas, cuidados com o armazenamento e regulamentações técnicas^(10)^. Aspectos importantes avaliados por este e outros estudos reforçam que há armazenamento e transporte inadequados de PPS esterilizados, falta de inspeção da embalagem antes do processo de esterilização e antes do uso, ausência de checagem da mudança de coloração do indicador químico de exposição, dentre outros^(2, 7, 10)^.

Portanto, estudos mais recentes referem a perda da esterilidade do PPS a eventos relacionados, e não a uma data específica^(2, 3, 7)^. A Resolução da Diretoria Colegiada (RDC) nº 15/2012, Art. 4, item VII, acata os princípios da esterilidade relacionada a eventos^(9)^. Porém, ao mesmo tempo que descreve a necessidade de um plano de avaliação da integridade das embalagens, não deixa claro como elaborar ou desenvolver esse plano, de modo que não há uniformidade nas condutas das diferentes instituições do país nem dos órgãos de fiscalização de cada região.

Após revisão aprofundada sobre o tema na literatura, verificou-se a ausência de um instrumento válido para avaliar eventos relacionados em embalagens de PPS esterilizados. Considerando que a manutenção da esterilidade depende de diversos fatores e que os meios para implementar esse princípio de segurança ainda são escassos, este estudo construiu e validou o instrumento Avaliação de Evento Relacionado à Manutenção da Esterilidade de Produtos para Saúde (AERMS, do inglês *Assessment of Event Related to Maintaining the Sterility*). Tal instrumento poderá integrar o plano de avaliação da integridade das embalagens e interromper o uso de PPS em situações de risco, especialmente daqueles críticos utilizados para procedimentos invasivos.

## OBJETIVO

Construir um instrumento e validar o conteúdo referente à avaliação de evento relacionado à manutenção da esterilidade de produtos para saúde processados pelo CME, tendo como aspecto norteador a integridade das embalagens de não tecido (SMS) e a de papel grau cirúrgico.

## MÉTODOS

### Aspectos éticos

O Comitê de Ética em Pesquisa aprovou o presente estudo, que atendeu às recomendações da Resolução nº 466/2012 do Conselho Nacional de Saúde. Os participantes assinaram o Termo de Consentimento Livre e Esclarecido (TCLE) em duas vias.

### Desenho, período e local do estudo

Trata-se de um estudo metodológico, que envolve o desenvolvimento de instrumentos. Esse desenho de estudo investiga métodos de obtenção, organização e análise dos dados, com base na elaboração, validação e avaliação de instrumentos e técnicas de pesquisa. Seu relato foi baseado no COnsensus-based Standards for the selection of health Measurement INstruments (COSMIN)^(11)^.

O estudo foi desenvolvido em duas etapas entre outubro de 2021 e dezembro de 2022. A primeira consistiu na construção do instrumento; e a segunda, em sua avaliação por um comitê de juízes para validade de conteúdo^(11, 12)^. O pré-teste foi desenvolvido em um hospital de ensino público, terciário/quaternário, de médio porte, localizado no interior do estado de São Paulo.

### População e Critérios de inclusão e exclusão

A população-alvo foi composta por profissionais de enfermagem atuantes no CME e nas unidades usuárias, com amostragem não probabilística e por conveniência. Os critérios são apresentados no Quadro 1.

**Quadro 1:** Critérios para seleção dos participantes. Brasil, 2021

	- Ser doutor e/ou mestre e/ou especialista na área específica. - Ter experiência prática e/ou ensino e/ou pesquisa de pelo menos três anos de atuação na área específica em território nacional, contemplando diferentes regiões do país, ou em território internacional (requer domínio da língua portuguesa). Além disso, deve preencher ao menos um dos seguintes: - Participar ou ter participado pelo menos dois anos em grupo de pesquisa na área. - Participar de eventos científicos relacionados ao tema. - Ter artigos publicados em revistas de referência.
CRITÉRIO 2	- Ser enfermeiro e/ou técnico de enfermagem na instituição de estudo. - Ter experiência prática de pelo menos três anos de atuação na área de enfermagem.

Para a validação de conteúdo, o instrumento construído foi submetido à apreciação de um comitê de juízes na área de interesse, para remoção, modificação ou acréscimo de itens^(11)^. Uma lista de nomes de profissionais com expertise no tema foi feita pela equipe de pesquisa, e a seleção foi realizada por análise do Currículo Lattes. Considerou-se que o comitê seria composto por 5 a 20 juízes; e o pré-teste, aplicado a uma amostra de 30 a 40 indivíduos da população-alvo^(12)^.

### Protocolo do estudo

#### Etapa 1: Construção do instrumento

A construção do instrumento baseou-se na literatura, diretrizes nacionais^(13, 14)^, internacionais^(7, 15, 16, 17)^, Resolução da Diretoria Colegiada – RDC nº 15, de 15 de março de 2012, que dispõe sobre as boas práticas do processamento de produtos para saúde no território nacional^(9)^, formação da equipe de pesquisa, protocolos da instituição, observação in loco dos processos de trabalho e experiência dos profissionais de enfermagem.

Como passo inicial, foi realizada uma busca nas bases de dados Bireme, PubMed, Embase, Cochrane, CINAHL e SciELO, para: a) estabelecer a estrutura conceitual; b) verificar instrumentos existentes; c) definir os objetivos do instrumento; e d) definir a população a ser envolvida, de modo a determinar o constructo de interesse e suas dimensões. Com isso, foram identificados os eventos-chave para composição do instrumento, tendo como princípio PPS embalados em SMS ou papel grau cirúrgico. A partir de então, foram construídos os itens e dimensões, a escala de resposta, a seleção e organização dos itens e a estruturação do instrumento^(12)^.

Uma versão preliminar do instrumento foi criada com auxílio de membros da equipe de enfermagem do CME da instituição onde foi realizado o estudo, utilizando a técnica de brainstorming, também conhecida como “chuva de ideias”, cujo objetivo é estimular a criatividade por meio da geração de um grande número de ideias sobre certo assunto^(18, 19)^. Ao total, foram três sessões de brainstorming, realizadas entre março e abril de 2022.

Em seguida, para verificar se os itens de cada domínio eram apropriados e se o instrumento poderia ser aplicado em diferentes realidades de hospitais públicos e privados, de baixa e alta demandas, foi realizada a validação de face com outras três instituições de saúde, utilizando a estratégia do benchmarking em um grupo focal^(20, 21, 22)^. O grupo focal aconteceu on-line, em uma única sessão, com duração de 60 minutos. As pesquisadoras apresentaram o instrumento aos participantes, depois foi dada a oportunidade para cada um expressar a sua opinião sobre a utilidade do instrumento no contexto do seu hospital, além de esclarecer dúvidas ou sugerir modificações. Com base nas sugestões, obteve-se a primeira versão do instrumento a ser enviada aos juízes. Foi também elaborado um guia de instruções para facilitar o preenchimento do instrumento, contendo as definições de cada um dos itens abordados.

#### Etapa 2: Validação de conteúdo

Os dados referentes à segunda etapa foram coletados entre junho e dezembro de 2022. A avaliação pelos juízes foi sequencial, individual e anônima, com feedback comentado, sendo o processo repetido até se atingir consenso. A carta-convite foi enviada via e-mail, considerando uma única tentativa de retorno. Foram avaliadas a clareza e pertinência dos itens, a abrangência das dimensões e a aparência geral do instrumento^(12)^. Para avaliá-lo, os juízes utilizaram uma escala tipo Likert de forma crescente, com pontuação de 1 a 4, na qual “1” significa “item não necessário ou não relevante/não claro/não abrangente” e “4” significa “item definitivamente essencial, muito relevante/muito claro/muito abrangente”^(20)^.

Após essa fase, foi realizado o pré-teste para avaliação semântica e prática. Os participantes receberam o instrumento, o guia de preenchimento e um formulário contendo a) dados pessoais para caracterização da amostra; b) avaliação geral sobre o instrumento (bom, regular ou ruim) e se as questões alinham-se com os valores e práticas profissionais na instituição; c) avaliação específica sobre a compreensão das instruções, dos itens do instrumento e das respostas, além de avaliar se cada item era considerado importante ou não; e d) tempo de preenchimento (início/término).

### Análise dos resultados e estatística

Os dados foram inseridos em uma planilha no Google Drive; e os resultados, analisados pelos pesquisadores entre cada rodada. Para medir a concordância entre os juízes, foram usados o Índice de Validade de Conteúdo (IVC), Razão de Validade de Conteúdo (RVC) e o coeficiente Kappa (k) modificado, desenvolvido para estudos de validade de conteúdo^(21)^. O IVC/RVC foi calculado pelo número de respostas “3” e “4” dividido pelo número total de respostas, indicando a proporção de juízes concordantes sobre determinados aspectos do instrumento e de seus itens, sendo considerado para este estudo um índice ≥ 0,80^(12)^. Para o *k* modificado, valores de 0,40 a 0,59 foram considerados razoáveis; de 0,60 a 0,74, bons; e acima de 0,74, excelentes^(23)^.

Os itens que não atingiram a pontuação em ambos os testes foram reformulados ou excluídos, e o instrumento foi submetido à avaliação dos juízes novamente, resultando no instrumento final após a segunda rodada. Para o pré-teste, utilizou-se estatística descritiva por meio de porcentagem, média, mediana, desvio-padrão (DP) e percentis 25 e 75 (Q1 e Q3).

## RESULTADOS

A versão preliminar do instrumento foi construída com 51 itens e cinco dimensões: 1) apresentação do produto, 2) evento relacionado, 3) selagem da embalagem, 4) indicador químico e 5) intercorrências.

Para a validação de face, foram convidados quatro enfermeiros de instituições públicas e privadas do estado de São Paulo e um técnico de enfermagem da instituição do estudo. Três enfermeiras compareceram ao grupo focal: uma de hospital privado e uma de hospital público de dois municípios do interior do estado de São Paulo; e uma enfermeira que trabalhava em dois hospitais, sendo um público e o outro privado de um município do estado do Rio de Janeiro. Duas enfermeiras têm especialização em gestão de CME, e uma tem experiência prática em CME superior a três anos, atendendo aos critérios de inclusão.

Após apresentação do instrumento, os participantes julgaram que os itens eram apropriados para avaliar a esterilidade relacionada a eventos de produtos para saúde esterilizados. Para que o instrumento pudesse ser aplicado no dia a dia, segundo a realidade de cada instituição, foi sugerido dividir a avaliação do produto em três sessões: “preparo”, “guarda e distribuição” e “a qualquer momento”, Foi usado o modelo de farol, com as cores verde, amarela e vermelha, respectivamente para cada sessão, dando origem à primeira versão do instrumento (material suplementar).

A validação de conteúdo transcorreu por um período de três meses entre o convite inicial aos juízes e a obtenção de consenso para todos os itens avaliados. A fim de compor o comitê de juízes, foram convidados 25 profissionais considerados elegíveis segundo os critérios estabelecidos. Obteve-se o retorno de dez deles: um recusou e nove aceitaram participar da pesquisa. Dentre os que aceitaram, cinco realizaram a avaliação do instrumento na primeira rodada, com taxa de resposta de 55,56% (5/9). Na segunda rodada, o instrumento reformulado foi entregue a estes cinco juízes, obtendo-se o retorno de quatro. Nesse caso, outros quatro profissionais receberam convite para integrar o comitê de juízes, dos quais um o aceitou. Sendo assim, a taxa de resposta da segunda rodada foi de 83,33% (5/6).

O comitê foi composto por cinco juízes brasileiros e um estrangeiro com domínio da língua portuguesa, sendo três do estado de São Paulo, um do Distrito Federal e dois residentes no exterior. Todos têm pós-graduação: um com título de mestre, dois com título de doutor e três com pós-doutorado. Quanto à área de atuação, três trabalham em órgãos regulamentadores: dois na área de ensino e pesquisa e um na assistência hospitalar, com tempo de experiência de 3 a 35 anos (Média = 17,33; DP = 11,33).

Após a primeira rodada, os itens “Embalagem: simples ou dupla”, “Etiqueta de identificação do preparo: sim ou não”, “O pacote a ser distribuído para as unidades apresenta”, e “Torção” foram excluídos. O item “Rubrica/Carimbo” foi reformulado para “Profissional responsável pela conferência”, O item “Marcações à caneta diretamente na embalagem” atingiu o valor esperado; entretanto, conforme sugestão dos juízes, foi realocado como evento na Dimensão 2, conforme sugestão dos juízes (Tabela 1). Todas as dimensões do instrumento foram consideradas abrangentes (IVC/RVC= 1,00; *k* modificado = 1,00).

**Tabela 1:** Análise da primeira rodada do instrumento Avaliação de Evento Relacionado à Manutenção da Esterilidade de Produtos para Saúde. Brasil, 2022

Sessões/Dimensões/Itens	Pertinência		Clareza	
	IVC*/RVC^†^	k^‡^	IVC*	*k* ^†^
Nº ordem	0,80	0,76	0,80	0,76
PREPARO	1,00	1,00	1,00	1,00
1. Apresentação do produto	1,00	1,00	0,80	0,76
Embalagem (simples/dupla)	0,60	0,42	1,00	1,00
Proteção em caso de PPS§ perfuro cortantes	1,00	1,00	0,80	0,76
Etiqueta de identificação do preparo contendo: nome do produto, número de peças, data do preparo, presença de indicador e nome do preparador	0,60	0,42	0,80	0,76
Etiqueta de identificação da esterilização contendo: nome do produto, número de peças, número do lote ou carga, data da esterilização, data-limite de uso, método de esterilização e nome do responsável pela esterilização	0,80	0,76	0,80	0,76
Marcações à caneta diretamente na embalagem	0,80	0,76	1,00	1,00
Rubrica/Carimbo	1,00	1,00	0,80	0,76
Data	1,00	1,00	0,80	0,76
GUARDA E DISTRIBUIÇÃO	0,80	0,76	1,00	1,00
2. Evento relacionado	0,80	0,76	1,00	1,00
O pacote a ser distribuído para as unidades apresenta:	0,60	0,42	1,00	1,00
Rasgo	0,80	0,76	1,00	1,00
Corte	0,80	0,76	1,00	1,00
Torção	0,60	0,42	0,80	0,76
Furos/Microfuros (olhar contra luz para grau cirúrgico)	1,00	1,00	1,00	1,00
Manchas na embalagem ou PPS^±^	1,00	1,00	0,80	0,76
Umidade na embalagem ou PPS^±^	1,00	1,00	0,80	0,76
Sujidade na embalagem ou PPS^±^	1,00	1,00	0,80	0,76
3. Selagem da embalagem	1,00	1,00	1,00	1,00
A selagem apresenta:	0,80	0,76	1,00	1,00
Falha na aderência	0,80	0,76	0,80	0,76
3.1. Para papel grau cirúrgico	1,00	1,00	1,00	1,00
A selagem apresenta:	1,00	1,00	1,00	1,00
Bolha	1,00	1,00	1,00	1,00
Delaminação	1,00	1,00	1,00	1,00
Queimadura	0,80	0,76	1,00	1,00
Dobra ou Vinco	1,00	1,00	1,00	1,00
4. Indicador químico	1,00	1,00	0,80	0,76
Corado	1,00	1,00	0,80	0,76
Ausente	1,00	1,00	0,80	0,76
Falha na coloração	1,00	1,00	0,80	0,76
Rubrica/Carimbo	0,80	0,76	0,80	0,76
Data	1,00	1,00	1,00	1,00
5. A qualquer momento	1,00	1,00	0,80	0,76
Intercorrências	1,00	1,00	1,00	1,00
Suspeita de que o pacote tenha sido aberto	1,00	1,00	1,00	1,00
Data-limite de uso expirada	1,00	1,00	1,00	1,00
Pacote caiu no chão	1,00	1,00	0,80	0,76
Reavaliação após queda	1,00	1,00	1,00	1,00
Rasgo	1,00	1,00	0,80	0,76
Corte	1,00	1,00	0,80	0,76
Torção	1,00	1,00	0,80	0,76
Furos/microfuros	1,00	1,00	1,00	1,00
Manchas	1,00	1,00	1,00	1,00
Umidade	1,00	1,00	0,80	0,76
Sujidade	1,00	1,00	1,00	1,00
Não se aplica	1,00	1,00	0,80	0,76
Rubrica/Carimbo	0,80	0,76	0,60	0,42
Data	1,00	1,00	0,80	0,76

*IVC – Índice de Validade de Conteúdo; ^†^RVC – Razão de Validade de Conteúdo; ^‡^k – Coeficiente Kappa modificado; ^§^PPS – produto para saúde.

Na avaliação do guia de preenchimento, as dimensões foram analisadas quanto à pertinência, clareza e abrangência (Tabela 2). A avaliação da aparência geral do guia de preenchimento obteve IVC = 0,80 e *k* modificado = 0,76, considerado excelente.

**Tabela 2:** Análise da primeira rodada do guia de preenchimento do instrumento Avaliação de Evento Relacionado à Manutenção da Esterilidade de Produtos para Saúde. Brasil, 2022

Dimensões	Pertinência		Clareza		Abrangência	
	IVC*/RVC^†^	*k* ^‡^	IVC*	*k* ^‡^	IVC^*^	*k* ^‡^
Apresentação do produto	1,00	1,00	0,80	0,76	0,80	0,76
Evento relacionado	1,00	1,00	1,00	1,00	1,00	1,00
Selagem da embalagem	1,00	1,00	1,00	1,00	0,80	0,76
Indicador químico	1,00	1,00	1,00	1,00	1,00	1,00
Intercorrências	1,00	1,00	1,00	1,00	1,00	1,00

*IVC – Índice de Validade de Conteúdo; ^†^RVC – Razão de Validade de Conteúdo; ^‡^k – Coeficiente Kappa modificado.

Com o reajuste dos itens, o instrumento deixou de ser subdividido em seções, ou seja, a apresentação da segunda versão constou em uma única coluna. Foi adicionado o item “Identificação do produto”, referente ao nome do produto em si ou a qualquer identificação contida na etiqueta, diferente do “Nº ordem”, que remete ao número de avaliações realizadas. Também foi adicionada a orientação “Avalie os itens de 2 a 5. Se identificar qualquer não conformidade, NÃO utilizar o pacote. Devolvê-lo ao CME para ser avaliado”, para nortear o avaliador de como proceder.

Após a formatação do instrumento, com alteração, exclusão e adição de itens, ele foi submetido a uma segunda avaliação pelos juízes, em que todos os itens e a aparência geral do instrumento obtiveram IVC/RVC ≥ 0,80 e *k* modificado ≥ 0,76, não havendo alteração de seu conteúdo e estrutura (material suplementar).

O pré-teste foi realizado de outubro a dezembro de 2022. Trinta profissionais de enfermagem participaram, sendo que 33,33% informaram ter ensino médio completo; 16,67%, ensino superior completo; e 50%, pós-graduação. Quanto à função na instituição, 56,67% eram técnicos de enfermagem e 43,33% eram enfermeiros, dentre os quais 20% ocupavam cargos na gerência de enfermagem. A média de idade foi de 42,97 anos (DP = 7,34) e do tempo de atuação na profissão foi de 16,03 anos (DP = 6,77). O instrumento foi testado em todo o hospital, sendo 33,33% no CME e 66,67% nas unidades usuárias (bloco operatório, unidades de terapia intensiva, unidade de internação, ambulatório e pronto atendimento), antes do uso do PPS ou em processos de auditoria interna.

O tempo de preenchimento variou de 1 a 13 minutos (Média = 4,33; DP = 2,95; Mediana = 4,00; Q1 = 2,00; Q3 = 5,00). Na avaliação geral do instrumento, 86,67% dos participantes consideraram-no bom, e 90,00% informaram haver alinhamento dele com os valores e práticas profissionais na instituição. Na avaliação específica dos itens, 86,67% concordaram parcialmente (20,00%) ou totalmente (66,67%) com a afirmação de que foi fácil compreender as instruções de preenchimento do instrumento e os itens do instrumento; e 90,00% concordaram parcialmente (26,67%) ou totalmente (63,33%) com a ideia de que foi fácil compreender e assinalar as respostas do instrumento.

Para os itens que os participantes assinalaram ter dificuldades para entender, estão os termos: Número de ordem, Delaminação, Queimadura, Vinco (túnel) e Reavaliação após a queda. Nesse caso, seguindo as sugestões apontadas, o termo “Nº ordem” foi substituído por “Quantidade de avaliações do produto”; em “Reavaliação após a queda”, foi acrescentado “Se sim, fazer a reavaliação após queda”; e, ainda nesse tópico, o item “Não se aplica” foi substituído por “Não houve dano à integridade da embalagem”, Para os demais itens apontados, averiguou-se que os participantes não consultaram o guia no momento do preenchimento.

Após o pré-teste, como não houve alteração no conteúdo, o instrumento não precisou retornar para avaliação dos juízes, sendo o conteúdo considerado válido para avaliação de evento relacionado à manutenção da esterilidade de produtos para saúde (material suplementar).

## DISCUSSÃO

As evidências científicas apontam que a esterilidade estará comprometida na ocorrência de eventos que causem dano à integridade da embalagem e da selagem^(7, 13, 14, 15, 16, 17)^. Em formato de checklist, o AERMS é um instrumento que pode ser aplicado a qualquer momento para avaliar a ocorrência de eventos relacionados nos produtos para saúde processados, interrompendo seu uso em situações de risco, especialmente os PPS críticos utilizados para procedimentos invasivos.

Durante a construção do instrumento, a contribuição ativa da equipe de enfermagem objetivou melhorar a aceitabilidade, relevância e a qualidade do que se pretendia avaliar, pois o que os pesquisadores consideram bom não necessariamente corresponde ao que é importante para os usuários do serviço^(24)^. Na área da saúde, o benchmarking é aplicado para medir e comparar resultados de outras instituições, com o intuito de implementar as melhores práticas existentes no mercado^(22)^. Inicialmente o AERMS foi construído para ser usado em diferentes etapas do processamento, desde o preparo do produto até seu uso no paciente, considerando checagens feitas por pessoas diferentes, em momentos diferentes, dentro de um mesmo formulário. Por meio da validação de conteúdo, percebeu-se que essa construção dificultaria sua implementação, além do que, em decorrência da exclusão de alguns itens, não haveria justificativa para manter os momentos de inspeção fragmentados. Em outras palavras, o instrumento pode adequar-se à realidade dos serviços, seja para inspeção dos PPS armazenados no CME ou nas unidades usuárias, seja para fins de auditoria ou em intercorrências com o PPS esterilizado, mas deve principalmente ser incorporado como uma prática rotineira e automática.

A validade de conteúdo é definida como o grau de relevância e representatividade dos itens abordados em relação ao propósito específico do instrumento^(21)^. Para esse tipo de validação, ainda não há consenso na literatura sobre o número de juízes que devem estar envolvidos^(1, 25)^. Porém, há uma recomendação para que haja, no mínimo, cinco pessoas para os procedimentos de análise estatística dos dados, levando-se em conta a característica e número de itens do instrumento, a formação, a experiência e a disponibilidade dos juízes sobre o que se pretende estudar^(11, 12)^. Embora nove pessoas elegíveis tenham aceitado participar do estudo, assinando o TCLE, apenas cinco retornaram com a avaliação do instrumento, sendo que os autores procuraram manter esse limite mínimo estabelecido nas duas rodadas. Ressalta-se que o processamento de produtos para saúde ainda é uma área escassa de especialistas, o que pode justificar a taxa de resposta obtida na avaliação do AERMS para fins de validação de conteúdo neste estudo.

Quanto aos itens excluídos, para “Embalagem: simples ou dupla”, entende-se que, a depender do tipo de embalagem, não é possível ao avaliador distinguir a presença de embalagens primárias e secundárias. Sobre a “etiqueta de identificação do preparo”, não são todos os serviços que adotam essa prática, porém ressalta-se que a RDC nº 15/2012, Art. 85, item VI, traz como obrigatório o nome do responsável pelo preparo^(9)^. O item “O pacote a ser distribuído para as unidades apresenta:” foi excluído pensando na aplicabilidade do instrumento a qualquer momento e não somente no momento da distribuição do produto pelo CME. Já o termo “Torção” foi excluído devido à sua ambiguidade com o conceito de dobra. Sobre esse evento, um estudo desenvolvido na França aponta para o risco de contaminação decorrente da dobra do papel grau cirúrgico na embalagem primária de produtos para saúde armazenados há pelo menos seis meses^(4)^.

No tocante aos itens que permaneceram, houve consenso sobre sua relevância e clareza, principalmente quanto à integridade da embalagem e manutenção da esterilidade. Ressalta-se que, embora seja defendida a mudança de paradigma de esterilidade referente ao tempo para esterilidade relacionada a eventos, o item “Data-limite de uso expirada” foi mantido já que se vivencia no Brasil um processo de transição e muitas instituições ainda utilizam o prazo de validade de esterilização recomendado pela RDC nº 15/2012^(9)^.

Esse prazo pode contribuir para o gerenciamento de estoque em vez do controle da perda de esterilidade do PPS. Um produto que fica inativo em estoque é desperdício de recurso financeiro que poderia ser investido em insumos necessários^(26)^. O processamento desnecessário de PPS devido ao prazo de validade expirado gera custos à instituição, desgaste da equipe, desgaste precoce dos próprios materiais, aumento da quantidade de resíduos de embalagens e desperdício de recursos naturais como água e energia elétrica. Um estudo realizado em um hospital terciário de Goa, na Índia, calculou que o custo total incorrido mensalmente para esterilizar PPS com prazo expirado foi de 8.772 rúpias indianas, o equivalente a cerca de 105 dólares nos dias atuais^(26)^. A gestão de materiais e recursos humanos no CME deve ser considerada como fator crítico no sistema de saúde, pois sua excelência operacional influencia diretamente os atendimentos aos usuários e pacientes. Está ligada aos objetivos do desenvolvimento sustentável (ODS), particularmente à ODS-3 Saúde e Bem Estar; ODS-9 Indústria, inovação e infraestrutura; e ODS-12 Consumo e produção responsáveis. Portanto, no contexto atual, o item “Data-limite de uso expirada” presente no AERMS pode ser utilizado como indicador de rotatividade do PPS, com base na implementação de um protocolo de inspeção.

Uma vez avaliado o instrumento e obtido o consenso entre os juízes, ele foi então submetido ao pré-teste, com aplicação em uma pequena amostra para verificar se todos os itens eram compreensíveis à população-alvo^(12)^. Trata-se de uma etapa importante no processo de pesquisa, que tem sido aplicada em outros estudos recentes de validação de conteúdo^(1, 27)^, pois ajuda os pesquisadores a identificarem potenciais problemas ou vieses durante a obtenção dos dados, assim como atestar a viabilidade de aplicação do instrumento no cotidiano da vida real. O pré-teste permitiu verificar um rápido preenchimento do AERMS, com percentuais de compreensão iguais ou superiores a 86% para os itens, estando o instrumento alinhado com os valores e práticas da instituição. Porém, observou-se que os participantes consultaram pouco o guia de preenchimento do instrumento, que era um documento separado e para o qual foi disponibilizado um exemplar por setor para consulta. Após o pré-teste, os autores optaram por manter as instruções no verso do instrumento, para uma consulta rápida sempre que necessário.

Portanto, ao utilizar o conceito de esterilidade relacionada a eventos, é necessário que as instituições de saúde estabeleçam boas práticas para que os PPS esterilizados sejam manuseados, transportados e armazenados com segurança, garantindo a integridade das embalagens e a diminuição do desperdício, carga de trabalho e custos^(7, 26, 28)^.

### Limitações do estudo

Uma limitação do estudo foram os autores optarem por não incluir no instrumento o tecido de algodão, devido à dificuldade no controle de qualidade de suas especificidades, tais como cerzimento, número de lavagens ou outras, especialmente quando da avaliação pelos usuários. Já outros tipos de embalagens como papel crepado e tyvek não são utilizados na instituição onde o estudo foi desenvolvido, portanto não foram avaliados. Contudo, os autores acreditam que os itens disponíveis no instrumento poderão ser úteis para avaliação dessas embalagens também.

### Contribuições para a área da Enfermagem

Considerando que a manutenção da esterilidade de um PPS depende de diversos fatores e que os meios para implementar esse princípio de segurança ainda são escassos, o presente estudo teve como contribuição a construção e validação de um instrumento para avaliar a ocorrência de eventos que podem comprometer a integridade das embalagens. O AERMS poderá ser útil para integrar o plano de avaliação da integridade das embalagens e mudar o paradigma de esterilidade relacionada a eventos para a prática assistencial. Isso auxiliará os gestores a avaliarem seus processos de trabalho tanto no CME quanto nas unidades usuárias e apoiará os profissionais de saúde envolvidos na assistência direta para decidir sobre o uso seguro do PPS, contribuindo para a segurança do paciente.

## CONCLUSÕES

A construção e validação de conteúdo do instrumento “*Assessment of Event Related to Maintaining the Sterility”* ou “AERMS”, para as embalagens SMS e papel grau cirúrgico, seguiu as recomendações presentes na literatura e está disponível para ser utilizado. Esse instrumento contribui para auxiliar no gerenciamento do processo de trabalho e na tomada de decisão sobre o uso seguro do produto para saúde.

## Data Availability

https://doi.org/10.25824/redu/KAXNUY
